# Skin exposure to soil microbiota elicits changes in cell-mediated immunity to pneumococcal vaccine

**DOI:** 10.1038/s41598-024-68235-8

**Published:** 2024-08-10

**Authors:** Marja I. Roslund, Noora Nurminen, Sami Oikarinen, Riikka Puhakka, Mira Grönroos, Leena Puustinen, Laura Kummola, Anirudra Parajuli, Ondřej Cinek, Olli H. Laitinen, Heikki Hyöty, Aki Sinkkonen

**Affiliations:** 1 Natural Resources Institute Finland, Luke, Viikki and Turku, Finland; 2 Faculty of Medicine and Health Technology, Tampere University, Arvo Ylpön Katu 34, 33520 Tampere, Finland; 3 Ecosystems and Environment Research Programme, Faculty of Biological and Environmental Sciences, University of Helsinki, Niemenkatu 73, 15140 Lahti, Finland; 4 Department of Medical Microbiology, 2nd Faculty of Medicine, Charles University, V Úvalu 84, Praha 5, 150 06 Prague, Czech Republic; 5 Fimlab Laboratories, Pirkanmaa Hospital District, Tampere, Finland; 6 Present Address: Department of Medicine, Karolinska Institutet, Huddinge, Sweden

**Keywords:** Immunology, Microbiology, Environmental sciences

## Abstract

A resilient immune system is characterized by its capacity to respond appropriately to challenges, such as infections, and it is crucial in vaccine response. Here we report a paired randomized intervention-control trial in which we evaluated the effect of microbially rich soil on immune resilience and pneumococcal vaccine response. Twenty-five age and sex matched pairs of volunteers were randomized to intervention and control groups. The intervention group rubbed hands three times a day in microbially rich soil until participants received a pneumococcal vaccine on day 14. Vaccine response, skin and gut bacteriome and blood cytokine levels were analyzed on days 0, 14 and 35. Peripheral blood mononuclear cells (PBMCs) were stimulated with vaccine components and autoclaved soil for cytokine production. Commensal bacterial community shifted only in the intervention group during the 14-day intervention period. When PBMCs collected on day 14 before the vaccination were stimulated with the vaccine components, IFN-y production increased in the intervention but not in the control group. On day 35, vaccination induced a robust antibody response in both groups. In parallel, gut bacterial community was associated with TGF-β plasma levels and TGF-β decrease in plasma was lower in the intervention group. The results indicate that exposure to microbially rich soil can modulate the cell-mediated immunity to components in pneumococcal vaccine.

## Introduction

Humans co-evolved with rich environmental microbiota. According to “Old Friends” hypothesis and biodiversity hypothesis, the interaction with microbes was essential to the evolution and development of resilient immune system^1,2^. Resilient immune system has the capacity to adapt to challenges, such as pneumococcus infections and more recently COVID-19, by developing and regulating an appropriate immune response^3^. Nowadays, exposure to diverse microbiota is diminished in urbanized societies, which is associated with the lack of immunological resilience^4–7^. In parallel, declined resilience of the immune system may increase the risk of infections, cancers, and vaccine failures^8^.

Many factors influence the immune response to vaccination, including genetics, age, sex, commensal microbiota, and numerous environmental factors^9^. Particularly, the elderly may suffer from severe infections and diminished efficacy of vaccines due to age-related weak resilience of the immune system. Vaccine responses may vary depending on geographical location and between developed and developing countries with different levels of daily exposure to rich microbiota and pathogens^9^. Importantly, there are indications that rural and urban populations may differ in their antibody responses to vaccinations: e.g. children living in rural areas have been reported to have higher antibody responses to tetanus vaccination^9^. As gut microbiome composition has been found to correlate with vaccine responses^10,11^, a potential cause for the different vaccine responses among populations living in different habitats stem from differences in gut microbiota. Studies have shown that particularly gut Ruminococcaceae is associated with cell-mediated immune responses, and cellular responses to oral vaccines^10,12^. Surprisingly, the effect of exposure to rich environmental microbiota on vaccine response has never been tested in a controlled intervention trial.

Studies comparing urban and rural populations have demonstrated that people who grow up in habitats characterized by plentiful contacts with microbially rich soil and diverse vegetation, such as traditional farms, are exposed to wide range of environmental microbes and tend to have a highly resilient immune system^7,13–16^. Evidence exists that environmental microbes transfer to the skin, and into the respiratory and gastrointestinal tracts of humans after soil exposure^4,17–19^. Based on this, we developed a protocol to test the effect of daily exposure to soil and its environmental microbiota on immune system resilience^20^. Our previous intervention trials demonstrated, for the first time, that an increased microbial exposure may indeed enhance immune regulation among daycare children^4,17,21^, and that greenness, i.e. vegetation coverage, does not explain the results^18^. We also observed how exposure to rich microbiota shifts gut Ruminococcaceae^4,22^ that includes several known and candidate probiotics^23,24^. In another study among adults, we demonstrated that exposure to natural plant and soil-based material is safe and could be used as an approach to modulate immune response^20,25^. However, there is no evidence whether this approach could affect the vaccine responses.

Infectious diseases are a significant cause of morbidity and mortality. In the United States, influenza and pneumonia represented the ninth and COVID-19 the third leading cause of deaths in the year 2020^26^. The highest pneumonia mortality rates are seen among elderly people^27^. The primary agent responsible for pneumococcal infections is *Streptococcus pneumoniae*. This bacterium is a common cause of respiratory and invasive infections. Pneumonia is a specific type of respiratory infection that can be caused by various pathogens, including bacteria, viruses, fungi, and other microorganisms. When caused by *Streptococcus pneumoniae*, it is referred to as pneumococcal pneumonia^28^. Globally, large numbers of vaccinated children are unprotected due to vaccine ineffectiveness, including 10 million children born each year, who are vaccinated but remain unprotected from pneumococcus^29^. Therefore, it is crucial to develop safe procedures to enhance vaccine response particularly among the elderly and other risk groups to prevent severe infections.

To address this need, we conducted a paired randomized controlled trial, in which we evaluated the effect of microbially rich soil on immune resilience and pneumococcal vaccine response. We hypothesized that exposure to microbially rich soil changes commensal skin and gut microbiota, improves the function of immune system and enhances immune response to vaccine components and to pneumococcal vaccination. Since the selected pneumococcal vaccine includes 13 serotypes of *Streptococcus pneumoniae* and a non-toxic variant of diphtheria toxin as adjuvant CRM197^30^, since three pathogenic *Corynebacterium* species are known to produce the diphtheria toxin^31^, and since soil is rich in non-pathogenic *Corynebacterium*, we were particularly interested in shifts in soil *Streptococcus* and *Corynebacterium* on skin, and their associations with immune response. In addition, based on previous studies^4,10,12,22^, we hypothesized that soil exposure shifts gut Ruminococcaceae community that is associated with cell-mediated immune responses.

## Results

### The function of immune system changed during the intervention

#### Cytokine and pneumococcal antibody levels in plasma

**Figure 1:**
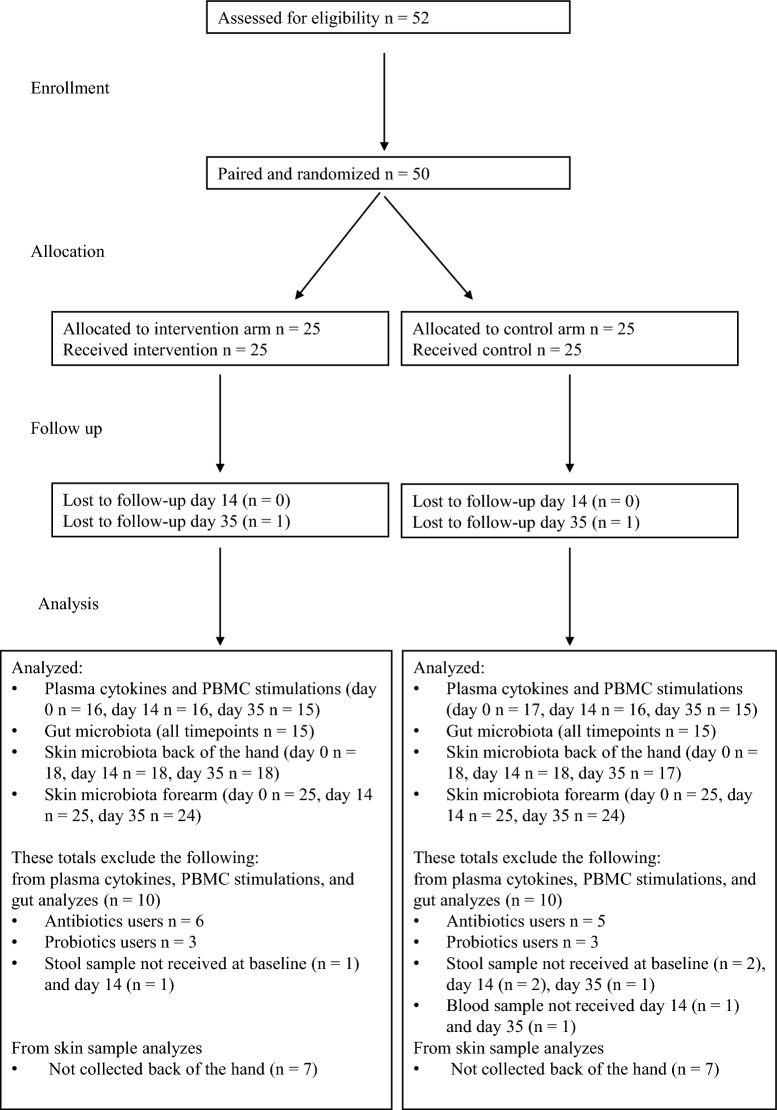
CONSORT diagram for study participants.

Twenty-five matched pairs were randomly assigned to the intervention and control groups. All the 25 intervention study subjects received intended treatment. After removing antibiotics and probiotics users there were 15 participants in each treatment (Fig. 1; Table 1, Table S1A–C), and twelve participants per treatment belonged to matched pairs.

**Table 1:** Characteristics of study participants. Age is presented as mean ± standard deviation. Outdoor recreations are presented at nominal scale (median ± confidence level 95% = cl): 1 = not at all, 2 = rarely, 3 = monthly, and 4 = weekly.

	Intervention	Control	Total
Gender, male	5	5	10
Gender, female	20	20	40
Gender, other	0	0	0
Age	56 ± 20	56 ± 19	56 ± 19
Dwelling type			
Detached house	2	2	4
Apartment building	22	22	44
Terraced house	1	1	2
Excluded from analyses*			
Antibioitcs users	6	5	11
Probiotics users	3	3	7
Pet ownership	5	5	10
Outdoor recreation			
Gardening	2 ± 0.56	2.5 ± 0.49	2 ± 0.37
Walking	4 ± 0.30	4 ± 0.23	4 ± 0.19
Cycling	3 ± 0.55	4 ± 0.42	4 ± 0.35
Hiking	2 ± 0.33	2 ± 0.33	2 ± 0.24
Berrypicking	2 ± 0.38	2 ± 0.38	2 ± 0.27
Mushroompicking	2 ± 0.41	2 ± 0.41	2 ± 0.29
Hunting	1 ± 0.26	1 ± 0	1 ± 0.12
Fishing	1 ± 0.41	2 ± 0.30	1 ± 0.25
Birdwatching	2 ± 0.53	2 ± 0.43	2 ± 0.35

*Study subjects using antibiotics and probiotics were excluded from the cytokine, PBMC stimulation, antibody and gut microbiome analyses. Living habits for the study subjects after excluded participants are showed in the Table S1.

**Figure 2:**
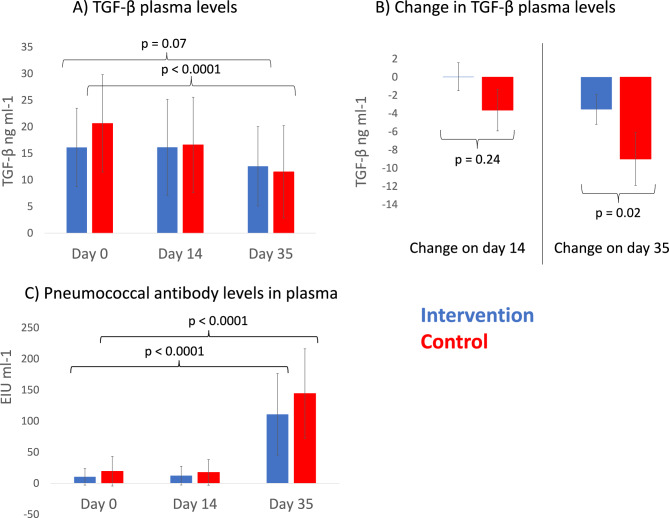
(**A**) TGF-β plasma concentrations (ng/ml) on day 0, 14 and 35. (**B**) TGF-β decreased more among study subjects in the control treatment group. (**C**) Pneumococcal antibodies [enzyme immunoassay units (EIU)] increased in both groups after the vaccination on day 35. P values are based on permutation tests with 5000 permutations.

Plasma TGF-β concentration decreased after pneumococcal vaccination in both intervention (p = 0.02 and permuted p = 0.07; Table S2A) and control groups (p < 0.0001; Fig. 2A; Table S2B). This decrease was more prominent in the control group (Change difference between groups: p = 0.02; Fig. 2B, Table S2C). No other differences were observed between treatment groups in plasma cytyokines (Table S2).

Pneumococcal vaccination induced a robust antibody response which did not differ between the intervention and control groups: tenfold increase (mean) was seen in the antibody levels in both groups (Fig. 2C). Prior to the vaccination, pneumococcal antibody levels stayed constant in both groups throughout the intervention. A trend for inverse correlation was seen between pneumococcal antibody levels and increasing age (p = 0.054; Fig. S1).

Pet ownership or outdoor recreation habits were insignificant in principal component analysis (Table S1B). All the study participants were of normal weight, i.e. body mass index was between 18.5 and 24.9. No special diets were reported in the questionnaires, and six study participants in each treatment received medications other than antibiotics (Table S1A).

#### The cell-mediated immune response of PBMCs stimulations

**Figure 3:**
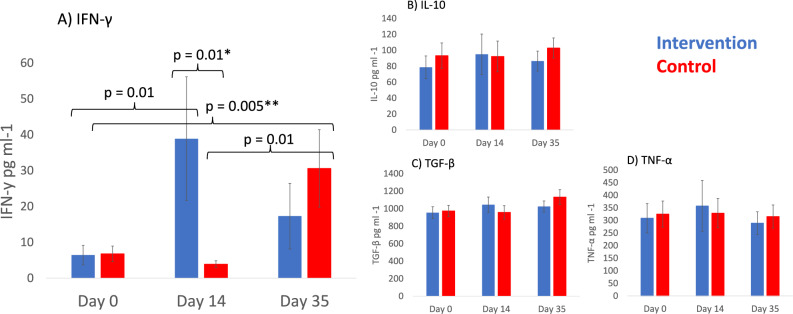
(**A**) IFN-γ, (**B**) IL-10, (**C**) TGF-β, and (**D**) TNF-α secretion (pg/ml) of PBMCs stimulated by pneumococcal vaccine components on day 0, 14 and 35 between intervention and control treatments. PBMCs were purified from blood samples collected on day 0, 14 and 35 among intervention and control treatment groups. Linear mixed models (LMM) were used to test for significant differences in time within and between treatments. *Change difference between treatments day 0 vs. 14, **Change difference between treatments day 14 vs. 35.

The cell-mediated immune response of PBMCs stimulated with components in pneumococcal vaccine did not differ between the groups at baseline (day 0). After the intervention period (day 14 before the vaccination), the IFN-γ response was significantly higher in the intervention than in the control group (Fig. 3A, Change difference between treatments LMM: p = 0.01; Table S3A). A contrasting difference was seen after pneumococcal vaccination: the response to pneumococcal vaccine components increased in the control group but not in the intervention group (Fig. 3A, change difference between treatments LMM p = 0.005; Table S3A). Despite visual impression IL-10, TGF-β, and TNF-α, there were no other differences between groups in cytokine responses in PBMC stimulations (Fig. 3B–D; Table S3A). Within intervention group, TNF-α response of CD3-CD28 stimulated PBMCs increased between days 0 and 14 (p = 0.04; Table S3B), whereas within the control group, TNF-α response of autoclaved soil stimulated PBMCs decreased between days 0 and 35 (p = 0.005; Table S3C).

### Intervention shifted the skin and gut bacterial composition

**Figure 4:**
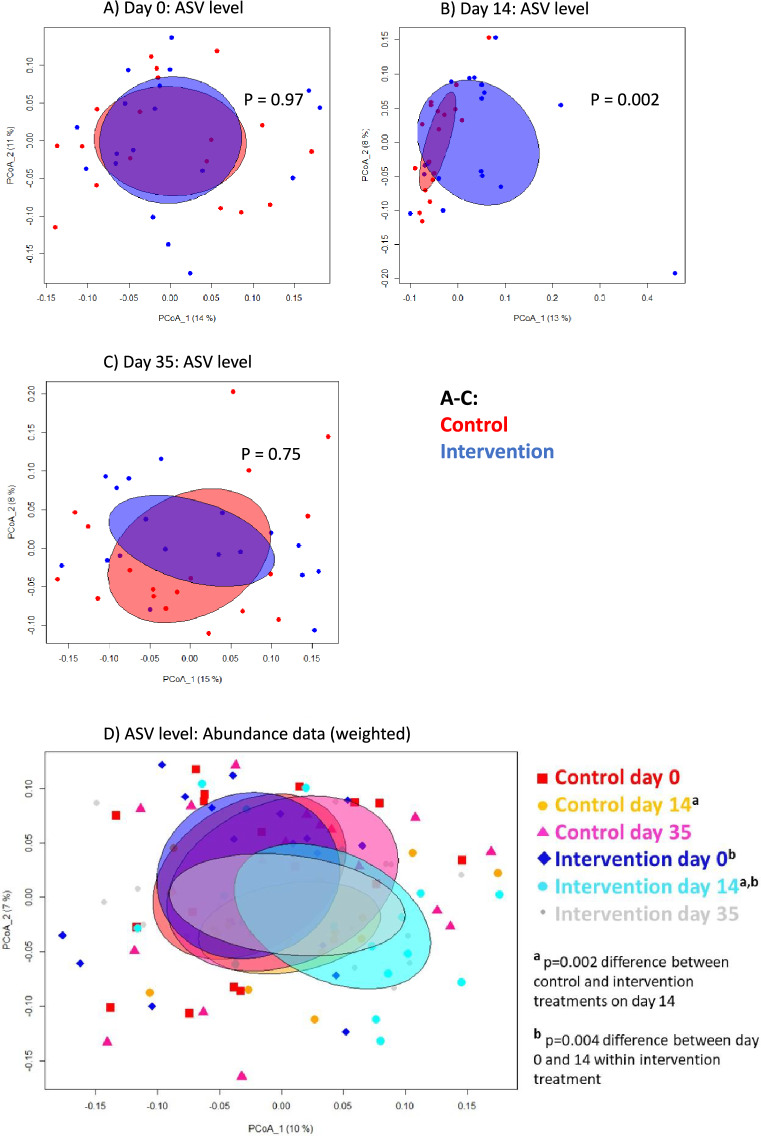
Principle coordinated analysis (PCoA) for skin (back of the hand) bacterial beta diversities between intervention and control group. PCoA plots are calculated with Euclidian distance at ASV level (abundance data i.e. weighted data) on (**A**) day 0, (**B**) day 14, and (**C**) day 35, and (**D**) for all days in a single plot. The differences between treatments and within treatments are tested using function *adonis2* in *vegan* package. Significance is based on permutation tests.

We first determined beta diversity, i.e., the shifts in skin microbial composition over time using permutational multivariate analysis of variance (PERMANOVA). Skin bacterial beta diversity changed markedly on the back of the hand during the 14-day exposure period in the intervention group (p = 0.004; Table S4D) while no clear change was seen in the control group (p = 0.22; Table S4E). In detail, beta diversity change occurred within main phyla Firmicutes, Proteobacteria, Actinobacteria and Bacteroidetes in the intervention group (p ≤ 0.002; Table S4D). Beta diversity was also notably different between intervention and control groups on day 14 (p = 0.002; Fig. 4B; Table S4B) while it did not differ between the groups on day 0 (Fig. 4A; Table S4A). On day 14, skin beta diversity differences between the groups included class Gammaproteobacteria (p = 0.005), order Lactobacillales (p = 0.001), family Thermoactinomycetaceae 1 (p = 0.02), and genus *Streptococcus* (p = 0.007) (Table S4B).

**Figure 5:**
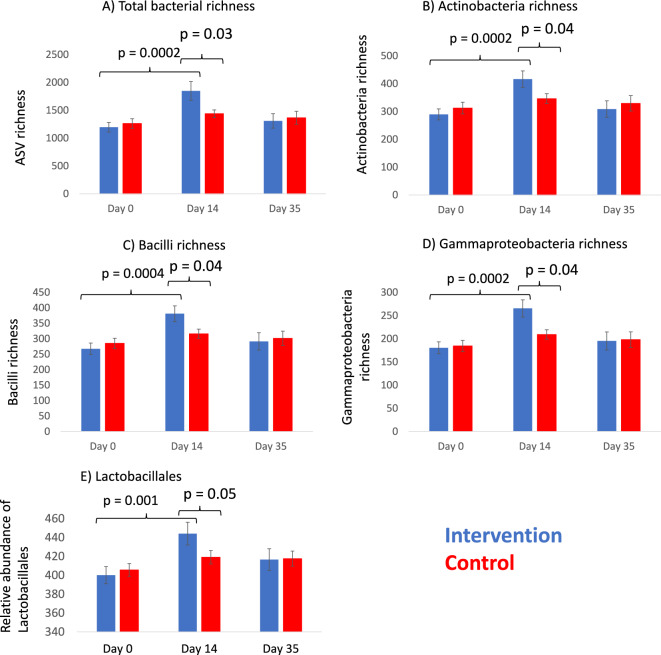
Richness of (**A**) total bacteria (ASV level), (**B**) Actinobacteria, (**C**) Bacilli, (**D**) Gammaproteobacteria, and (**E**) relative abundance (max. 7334) of order Lactobacillales on the back of the hand on day 0, 14 and 35 in intervention and control groups. Significance is based on 5000 permutations.

To further investigate temporal shifts in skin bacterial variables (richness, relative abundance and alpha diversity), we constructed LMM models taking the pairing of intervention and control participants into account. Changes that differed between intervention and control groups during the 14-day exposure period included an increase in the richness of total bacteria, particularly Gammaproteobacteria, Actinobacteria (p = 0.0002), and Bacilli (p = 0.0004) that was seen on the back of the hand in the intervention group (Fig. 5A–D; Table S5A). Similarly, the relative abundance of order Lactobacillales on the back of the hand increased only in the intervention group (p = 0.001; Fig. 5E; Table S5B). Altogether 31 genera, including *Corynebacterium* (p = 0.001), increased on the back of the hand among intervention study subjects during the 14-day intervention period and these shifts differed significantly compared to the control group (Table S5C). Sequence data included 34,125 *Corynebacterium* ASVs of which 11 increased on the back of the hand of intervention study subjects (Table S6). According to BLAST results these ASVs are mostly uncultured bacterium (Table S6). Total bacterial richness increased also on the forearm among intervention subjects (p = 0.02). In the control group no such change was seen (P = 0.7). No other differences between treatments were observed in the forearm bacterial community (data not shown).

On day 35, skin beta diversity of Thermoactinomycetaceae 1 (p = 0.04) and unclassified bacterial genera within Alphaproteobacteria (p = 0.007) still differed between treatment groups (Table S4C). Among intervention subjects, the relative abundance of Thermoactinomycetaceae 1 on the back of the hand was higher compared to the control group (p = 0.02). Other differences were no longer observed on day 35.

On day 14, a clear change was seen in gut bacterial communities in the intervention group as the relative abundance of family Thermoactinomycetaceae 1, particularly genus *Thermoactinomyces* (p = 0.003) and genus *Conexibacter* (p = 0.009) increased (Table 2A). Altogether 10 genera increased in the gut among intervention study subjects during the 14-day intervention period (p ≤ 0.04; Table 2A). No such changes were seen in the control group (p > 0.25; Table 2B). Richness of gut Ruminococcaceae (day 35: p = 0.03), particularly unclassified genera within Ruminococcaceae (day 14: p = 0.04 and day 35: p = 0.01), showed a decreasing trend in the intervention group compared to control group during the study period (Table 2C). Other changes in the gut were not observed on day 35.

**Table 2:** Linear mixed model (LMM) results within (A) intervention and (B) control group for gut bacteria changes at family and genus level, and (C) gut bacterial richness changes between treatment groups for Ruminococcaceae.

	Day 0		Day 14		Day 35		LMM: Day 0 vs Day 14			LMM: Day 0 vs Day 35		
	Mean	sd	Mean	sd	Mean	sd	t	p	Perm.p	t	p	Perm.p
(A) Within intervention group												
Family Thermoactino-mycetaceae 1	1.4	2.0	1.9	2.0	1.4	2.0	3.0	**0.003**	**0.008**	0.2	0.82	0.81
Genus Thermoactinomyces	1.4	2.0	1.9	2.0	1.4	2.0	3.0	**0.003**	**0.005**	0.2	0.82	0.81
Family Conexibacteraceae	1.1	2.2	1.5	2.2	1.1	2.1	2.6	**0.009**	**0.012**	0.2	0.82	0.82
Genus Conexibacter	1.1	2.2	1.5	2.2	1.1	2.1	2.6	**0.009**	**0.012**	0.2	0.82	0.82
Family Clostridiales Incertae Sedis III	1.1	2.2	1.5	2.3	1.1	2.1	2.5	**0.012**	**0.016**	0.5	0.64	0.62
Genus Methylophilaceae unclassified	1.1	2.2	1.7	2.4	1.3	2.2	2.5	**0.013**	**0.013**	0.9	0.35	0.35
Family Clostridiales Incertae Sedis XII	1.2	2.1	1.5	2.2	1.1	2.1	2.3	**0.023**	**0.027**	-0.4	0.69	0.69
Genus Fusibacter	1.2	2.1	1.5	2.2	1.1	2.1	2.3	**0.023**	**0.031**	-0.4	0.69	0.70
Genus Dorea	7.4	9.8	8.5	10.4	7.2	9.7	2.2	**0.028**	**0.034**	0.4	0.70	0.70
Genus Dermacoccus	3.9	6.2	4.5	6.5	3.9	6.0	2.1	**0.032**	**0.038**	0.7	0.46	0.47
Genus Neisseria	9.7	11.7	11.2	11.9	10.3	10.9	2.1	**0.033**	**0.043**	1.1	0.29	0.30
Genus Rhodospirillales unclassified	7.6	4.4	8.1	4.7	7.7	4.3	2.1	**0.036**	**0.040**	1.1	0.26	0.27
Genus Methylobacterium	3.3	6.5	3.8	6.8	3.3	6.4	2.0	**0.042**	**0.050**	0.6	0.57	0.58
Genus Ligilactobacillus	1.2	2.1	1.6	2.3	1.2	2.1	2.0	**0.043**	**0.046**	0.6	0.58	0.57
Family Methylophilaceae	2.4	4.3	3.0	4.5	2.3	4.2	1.9	0.051	0.061	0.3	0.77	0.76
Genus Kocuria	8.7	14.6	10.0	15.3	8.6	14.3	1.9	0.052	0.054	0.2	0.80	0.81
Genus Psychrobacter	1.2	2.1	1.3	2.3	1.1	2.1	1.9	0.053	0.061	0.4	0.72	0.72
(B) Within control group												
Family Thermoactino-mycetaceae 1	1.4	2.0	1.3	1.8	1.2	1.7	− 0.7	0.50	0.45	− 1.0	0.31	0.30
Genus Thermoactinomyces	1.4	2.0	1.3	1.8	1.2	1.7	− 0.7	0.50	0.47	− 1.0	0.31	0.28
Family Conexibacteraceae	0.9	1.8	0.9	1.9	1.0	1.8	− 1.0	0.31	0.32	− 0.6	0.56	0.55
Genus Conexibacter	0.9	1.8	0.9	1.9	1.0	1.8	-1.0	0.31	0.31	-0.6	0.56	0.54
Family Clostridiales Incertae Sedis III	0.8	1.8	1.0	1.9	0.9	1.8	-0.2	0.88	0.86	-0.7	0.49	0.49
Genus Methylophilaceae unclassified	0.9	1.8	0.9	1.9	0.8	1.8	-1.1	0.25	0.25	-1.6	0.10	0.12
Family Clostridiales Incertae Sedis XII	0.8	1.8	0.9	1.9	0.8	1.8	-0.6	0.57	0.57	-1.1	0.27	0.28
Genus Fusibacter	0.8	1.8	0.9	1.9	0.8	1.8	-0.6	0.57	0.57	-1.1	0.27	0.27
Genus Dorea	6.5	8.0	7.0	8.3	6.2	8.2	-0.6	0.55	0.55	-1.4	0.15	0.15
Genus Dermacoccus	3.3	5.2	3.7	5.3	3.5	5.2	-0.4	0.67	0.67	-0.9	0.37	0.38
Genus Neisseria	7.1	9.8	8.9	9.8	7.4	10.0	0.2	0.86	0.91	-1.2	0.22	0.21
Genus Rhodospirillales unclassified	6.6	3.8	6.9	3.7	6.8	4.0	-0.3	0.79	0.75	-0.5	0.64	0.61
Genus Methylobacterium	2.5	5.4	2.8	5.6	2.5	5.5	-0.9	0.37	0.37	-1.3	0.18	0.18
Genus Ligilactobacillus	0.7	1.8	0.9	1.9	0.8	1.8	-0.5	0.58	0.58	-1.1	0.27	0.29
Family Methylophilaceae	1.6	3.6	1.9	3.7	1.6	3.7	-0.6	0.57	0.56	-1.4	0.18	0.18
Genus Kocuria	7.5	11.9	8.6	12.1	7.4	12.1	-0.3	0.79	0.77	-1.2	0.22	0.23
Genus Psychrobacter	0.7	1.8	0.9	1.9	0.8	1.8	-0.5	0.58	0.57	-1.1	0.27	0.30

(C) Between treatment groups	Intervention			Control			LMM: Between treatments					
	Day 0	Day 14	Day 35	Day 0	Day 14	Day 35	Day 0 vs Day 14			Day 0 vs Day 35		
	Mean ± sd	Mean ± sd	Mean ± sd	Mean ± sd	Mean ± sd	Mean ± sd	t	p	Perm.p	t	p	Perm.p
Family Ruminococcaceae richness	16.7 ± 5.8	17.3 ± 4.4	14.6 ± 6.0	15.7 ± 3.7	17.4 ± 3.9	17.7 ± 3.9	-0.68	0.497	0.518	-2.2	**0.030**	**0.034**
Genus Ruminococcaceae unclassified richness	10.6 ± 4.1	11.2 ± 3.8	8.9 ± 4.0	8.0 ± 1.6	9.7 ± 2.7	9.7 ± 2.2	2.01	**0.044**	0.415	-2.6	**0.010**	**0.014**

Data is presented as mean ± standard deviation (sd). LMM statistics are reported as t value, probability p value, permuted p value (5000 permutations). Significant p values are in bold.

#### Gut beta diversity was associated with TGF-β in plasma

To investigate if the gut bacterial community are linked to the immune balance, we used principal coordinates analysis (PCoA) to score the bacterial amplicon sequence variants (ASVs) onto an ordination and assessed correlation with immune markers with *Envfit* function in R. TGF-β concentration in plasma was associated with beta diversity of gut bacterial community (p = 0.001) on day 35 when all study subjects were analyzed in the same model (Table 3A; Fig. S2). When intervention and control study subjects were analyzed separately, the association between plasma TGF-β and gut beta diversity was observed only in the intervention group on day 35 (p = 0.03; Table 2B). In addition, on day 35, beta diversity of gut Ruminococcaceae was associated with IL-10 (p = 0.004) and TNF-α (p = 0.04) response of pneumococcal vaccine stimulated PBMCs only in the intervention group (Table 3B). These associations were observed also from autoclaved soil stimulated PBMCs within the intervention group (Table 3B). No such responses were observed within the control group (p > 0.2; Table 3C).

**Table 3:** Correlation between beta diversity of gut bacteria and TGF-β in plasma and the production of IL-10 and TNF-α from pneumococcal vaccine and autoclaved soil stimulated PBMCs at day 0, 14 and 35.

Total bacterial beta diversity	Day 0		Day 14		Day 35	
	R2	p value	R2	p value	R2	p value
(A) Enfvit analyses with all study participants						
TGF-β in plasma	0.009	0.942	0.013	0.874	0.586	**0.001**
IL-10, PBMCs stimulated with vaccine	0.067	0.616	0.009	0.897	0.197	0.186
TNF-α, PBMCs stimulated with vaccine	0.045	0.739	0.064	0.481	0.216	0.186
IL-10, PBMCs stimulated with autoclaved soil	0.027	0.807	0.025	0.797	0.072	0.615
TNF-α, PBMCs stimulated with autoclaved soil	0.146	0.222	0.013	0.883	0.003	0.985
Ruminococcaceae beta diversity						
TGF-β in plasma	0.015	0.916	0.199	0.171	0.080	0.547
IL-10, PBMCs stimulated with vaccine	0.084	0.596	0.011	0.927	0.172	0.268
TNF-α, PBMCs stimulated with vaccine	0.109	0.461	0.035	0.802	0.308	0.070
IL-10, PBMCs stimulated with autoclaved soil	0.053	0.698	0.020	0.923	0.196	0.212
TNF-α, PBMCs stimulated with autoclaved soil	0.014	0.865	0.032	0.779	0.410	**0.015**
(B) Enfvit analyses within intervention group						
TGF-β in plasma	0.161	0.695	0.204	0.451	0.443	**0.030**
IL-10, PBMCs stimulated with vaccine	0.364	0.270	0.150	0.551	0.287	0.162
TNF-α, PBMCs stimulated with vaccine	0.166	0.630	0.037	0.766	0.200	0.292
IL-10, PBMCs stimulated with autoclaved soil	0.032	0.933	0.017	0.891	0.183	0.254
TNF-α, PBMCs stimulated with autoclaved soil	0.520	0.188	0.261	0.361	0.216	0.258
Ruminococcaceae beta diversity						
TGF-β in plasma	0.453	0.166	0.072	0.739	0.143	0.524
IL-10, PBMCs stimulated with vaccine	0.405	0.244	0.385	0.173	0.751	**0.004**
TNF-α, PBMCs stimulated with vaccine	0.216	0.530	0.102	0.474	0.542	**0.039**
IL-10, PBMCs stimulated with autoclaved soil	0.047	0.834	0.067	0.641	0.579	**0.041**
TNF-α, PBMCs stimulated with autoclaved soil	0.268	0.268	0.570	**0.030**	0.589	**0.022**
(C) Enfvit analyses within control group						
TGF-β in plasma	0.032	0.797	0.223	0.445	0.072	0.852
IL-10, PBMCs stimulated with vaccine	0.012	0.927	0.112	0.744	0.287	0.523
TNF-α, PBMCs stimulated with vaccine	0.044	0.886	0.591	0.129	0.391	0.371
IL-10, PBMCs stimulated with autoclaved soil	0.154	0.590	0.449	0.186	0.026	0.940
TNF-α, PBMCs stimulated with autoclaved soil	0.298	0.451	0.371	0.188	0.076	0.878
Ruminococcaceae beta diversity						
TGF-β in plasma	0.197	0.552	0.502	0.129	0.325	0.488
IL-10, PBMCs stimulated with vaccine	0.636	0.112	0.366	0.235	0.353	0.329
TNF-α, PBMCs stimulated with vaccine	0.515	0.171	0.319	0.332	0.568	0.235
IL-10, PBMCs stimulated with autoclaved soil	0.588	0.133	0.029	0.908	0.109	0.955
TNF-α, PBMCs stimulated with autoclaved soil	0.395	0.304	0.113	0.781	0.019	0.932

Analyses were done with (A) all study participants, and (B) within intervention and (C) control treatment groups separately. Beta diversity is reported for total bacterial community level (ASV) and for family Ruminococcaceae. Correlation was assessed using function envfit function in R and statistics are reported as the square of the correlation (R2) and probability p value. Significance is tested by permutation test. Significant values are in bold.

#### Skin beta diversity was associated with IFN-γ production from soil stimulated PBMCs

On the day 14, skin total (back of the hand) (p = 0.03), Gammaproteobacteria (p = 0.03), Bacteroidia (p = 0.03) and order Lactobacillales (p = 0.04) beta diversities correlated with IFNγ (R_2_ = 0.56) production and TGF-β:IFNγ ratio (R_2_ = 0.54, p ≤ 0.018) from autoclaved soil stimulated PBMCs when both treatment groups were analyzed together (Table S4A). When treatment groups were analyzed separately, the associations with TGF-β:IFN-γ ratio were observed moderately only among intervention study subjects (p ≤ 0.06; Table 4B). No correlations were seen between skin microbial communities and immune markers in plasma.

**Table 4:** Correlation between beta diversity of bacteria on the back of the hand and the production of interferon-gamma (IFN-γ) and IFN-γ and transforming growth factor-beta (TGF-β) ratio from autoclaved soil stimulated PBMCs at day 0, 14 and 35. Analyses were done with (A) all study participants, and (B) within intervention and (C) control treatment groups separately.

Total bacterial beta diversity	Day 0		Day 14		Day 35	
	R2	p value	R2	p value	R2	p value
(A) Enfvit analyses with all study participants						
IFN-γ	0.389	0.203	0.562	**0.030**	0.376	0.165
TGF-β:IFN-γ	0.198	0.435	0.537	**0.018**	0.165	0.369
Gammaproteobacterial beta diversity						
IFN-γ	0.388	0.212	0.562	**0.032**	0.360	0.162
TGF-β:IFN-γ	0.250	0.348	0.537	**0.015**	0.128	0.462
Bacteroidia beta diversity						
IFN-γ	0.325	0.244	0.562	**0.026**	0.415	0.131
TGF-β:IFN-γ	0.151	0.521	0.537	**0.018**	0.175	0.313
Lactobacillales beta diversity						
IFN-γ	0.407	0.168	0.562	**0.037**	0.381	0.149
TGF-β:IFN-γ	0.190	0.428	0.537	**0.013**	0.415	0.146
(B) Enfvit analyses within intervention group						
IFN-γ	0.510	0.242	0.564	0.085	0.473	0.252
TGF-β:IFN-γ	0.315	0.463	0.532	0.060	0.150	0.657
Gammaproteobacterial beta diversity						
IFN-γ	0.692	0.200	0.564	0.115	0.522	0.256
TGF-β:IFN-γ	0.285	0.529	0.532	0.057	0.072	0.853
Bacteroidia beta diversity						
IFN-γ	0.556	0.238	0.564	0.100	0.458	0.302
TGF-β:IFN-γ	0.225	0.603	0.532	**0.049**	0.268	0.462
Lactobacillales beta diversity						
IFN-γ	0.498	0.358	0.565	0.086	0.470	0.290
TGF-β:IFN-γ	0.278	0.562	0.532	0.057	0.415	0.146
(C) Enfvit analyses within control group						
IFN-γ	0.398	0.833	0.095	0.817	0.998	0.125
TGF-β:IFN-γ	0.663	0.667	0.911	0.133	0.991	0.167
Gammaproteobacterial beta diversity						
IFN-γ	0.505	0.750	0.820	0.317	0.989	0.125
TGF-β:IFN-γ	0.500	0.750	0.877	0.225	0.724	0.583
Bacteroidia beta diversity						
IFN-γ	0.188	0.958	0.382	0.608	0.987	0.167
TGF-β:IFN-γ	0.482	0.792	0.621	0.408	0.697	0.583
Lactobacillales beta diversity						
IFN-γ	0.513	0.750	0.063	0.867	0.955	0.167
TGF-β:IFN-γ	0.640	0.625	0.046	0.908	0.934	0.417

Beta diversity was assessed at total bacterial community level (ASV), and for Gammaproteobacteria, Bacteroidia and Lactobacillales. Correlation was assessed using function envfit function in R and statistics are reported as the square of the correlation (R2) and probability p value. Significance is tested by permutation test. Significant values are in bold.

## Discussion

As far as we are aware of, our findings are the first to suggest that exposure to microbially rich and diverse soil modulates vaccine response, in addition to posing shifts in commensal microbiota and immune system function. Five findings support the idea that the intervention had an immunological effect. First, plasma TGF-β decreased more in the control group compared to the intervention group. Second, gut beta diversity was associated with plasma TGF-β concentration particularly among the intervention study subjects. Third, gut Ruminococcaceae that shifted among intervention study subjects on day 35 was associated with cytokine responses from PBMCs stimulated with pneumococcal vaccine or autoclaved soil only within the intervention treatment group. Fourth, IFN-γ secretion by pneumococcal antigen stimulated PBMCs was different between the study groups on day 14 before the vaccination. Finally, immune responses, such as the TGF-β and IFN-γ, were associated with skin and gut microbiota among intervention study subjects.

The fact that in the intervention group cytokine secretion by pneumococcal vaccine and autoclaved soil stimulated PBMCs correlated with gut and skin bacterial beta diversity supports the hypothesis that soil exposure affects cell-mediated immune response. The mechanisms of this effect are not known, but a potential candidate is the stimulation of pre-existing immunity by diverse pneumococcal-like bacteria in soil. This is supported by the findings that the beta diversity and relative abundance of skin Lactobacillales and the beta diversity of skin *Streptococcus* shifted among the intervention study subjects only. As pneumococci (*Streptococcus pneumonia*) belong to the order Lactobacillales, the immune system of study subjects in the intervention group plausibly had to cope with non-infectious soil bacteria reminiscent to infectious pneumococci during the intervention.

Since antigen stimulation was done with Prevenar 13 vaccine including a non-toxic variant of diphteria toxin, antigen stimulation measured the response to pneumococcal capsular polysaccharides together with diphtheria antigen conjugated to vaccine. Soil used in this study did not include *Corynebacterium* known to produce diphtheria toxin^31^, however, soil contained over 20,000 environmental *Corynebacterium* ASVs. Since the relative abundance of several unidentified uncultured *Corynebacterium* ASVs became a magnitude higher on the skin of the intervention study subjects only, an alternative explanation for different cell-mediated immune responses between treatment groups might be that rich soil *Corynebacterium* community modulated immune response via their endogenous diphtheria toxin homologs.

Since IFN-γ is a proinflammatory cytokine that has important role in innate and adaptive immunity, the finding that IFN-γ secretion by pneumococcal antigen stimulated PBMCs was different between groups is interesting. Aging in general is associated with poor vaccine responses and multiple defects in the ability to produce IFNs in response to infection^32^. Because the current soil exposure was associated with higher production of IFN-γ, soil exposure could be helpful for an aging immune system to produce an effective response to pneumococcal and potentially other vaccines. Even more, since IFN-γ has disease-protective activities due to its dual role, pro- as well as anti-inflammatory^33^, daily exposure to microbially rich soil extract could also devise better therapeutic approaches in preventing autoimmune diseases, providing that safety issues are taken into account. However, based on the current study findings, it is not yet possible to say whether the intervention was beneficial for vaccine response or not.

The finding that the TGF-β in plasma is downregulated three weeks after vaccination is intriguing, because TGF-β induces the differentiation of naïve T cells in T helper type 17 (Th17) that are important in vaccine-induced immunity^34^. The downregulation might follow ceasing immediate immune reactions triggered by pneumococcal vaccination. Thus, the difference between groups in TGF-β decrease may indicate slightly different timeframe for the immune response. Since butyrate can induce TGF-β production^35^, a reason for lower decrease in TGF-β among intervention group compared to control group might be higher production of butyrate among intervention subjects. Indeed, evidence exists that soil butyrate-producers may aid to supplement the human gut bacterial community^36,37^. The idea is supported by the finding that gut Ruminococcaceae including butyrate-producing bacteria was altered in the intervention group, and gut bacterial community was associated with TGF-β plasma concentration on day 35 among the intervention study subjects. Even though we did not study potential health benefits of butyrate^38^, the finding is intriguing, particularly because similar shifts in gut Ruminococcaceae community have earlier been found in intervention trials with daycare children^4^ and soil-exposed piglets^39^, and while urban dwellers and rural inhabitants have been compared^22^.

Interestingly, relative abundance of Thermoactinomycetaceae 1 increased on the skin and in the gut of the intervention study subjects. This may be important for potent memory effector responses; in our earlier intervention trial with daycare children, the increase in skin Thermoactinomycetaceae 1 relative abundance was associated with higher total and memory T_reg_ cell frequencies^18^. In the earlier trial, daycare children were exposed to sandbox sand that was enriched with the same soil mixture as in the current study^40^. Thus, the results of the current and the previous study^18^, and the metagenomic data of the soil material^40^ show together that this bacterial family is transferred from the soil to the hands and into the gastrointestinal tract.

The current trial used matched-pair randomization. This underlines the robustness of our findings. One limitation of this study is the absence of interval samples between the day 14 and 35. It would have been interesting to analyze if the vaccine response occurred faster in the intervention group compared to the control group. The robust response become evident in both treatments by day 35, indicating that the vaccine is effective within 20 days. It was fascinating to recognize potential differences in all measured cytokines in PBMC stimulation with Prevenar 13 vaccine components (Fig. 3A–D) in the end of the intervention period; in a larger trial the high level of between individual variation in immune response among urban dwellers might not prevent the statistically significant findings. As this study was not a placebo-controlled trial, we cannot exclude the potential effect of daily routines per se. However, as our previous daycare trial was placebo-controlled^18^, immune modulation and microbial shifts were similar with the current study, we consider a major role of daily routines unlikely. Further, we have seen similar responses in mouse studies^41,42^. Despite this, we encourage the planning of double-blinded trials and a dense sampling interval after vaccination. We found an increase in cell-mediated immunity in the intervention group before the vaccination. However, at the day 35 after vaccination this response had disappeared. These results might indicate that the 14-day intervention was not protective after three weeks, but in contrast with this idea a significant reduction in TGF-β plasma levels was observed only in the control group. Thus, future studies must dig out if there is a time window of beneficial response, and whether the potentially non-protective visual reduction in TGF-β plasma levels were consequences of exposure’s shortness. The results of the current study support our hypothesis that exposure to microbially rich soil changes immune response to vaccine components, but it cannot be stated whether and how microbial exposure enhances vaccine response, i.e. antibody levels. The total vaccine antibody level was used to assess the vaccine response since we assumed that the intervention would not have different effects on serotype-specific responses but would cause a more holistic effect by stimulating the immune system via pattern recognition receptors. However, the current study encourages research on the role microbial exposure in vaccine response, which is a novel research area in immunology. Larger studies and different exposure routes are advantageous in future research focusing on the enhancement of vaccine response. Importantly, in the current study, the response to PBMC stimulation was different in the intervention and control arms, and the shift in TGF-β plasma concentration was different after the vaccination.

The finding that environmental biodiversity may boost vaccine response is of utmost importance. More than half of human population are expected live in urban areas by the year 2050^43^, and many of them lack access to natural or semi-natural green space. Our findings hence contribute to scientific debate about future practices in modern urban planning and landscaping in both developed and developing countries. Restoring environmental biodiversity improve urban soil microbiota and quality^44–46^, and can thus be an effective public health intervention and also ameliorate COVID-19 recovery^47^. Where urbanites cannot access natural biodiversity on a daily basis, the current study paves the way for research that aims at modifying urban living environments outdoors^48^ and indoors^49,50^.

## Conclusions

The impact of soil microbiota on the immune system and consequent vaccine response, as well as disease risk, are complex. We have shown that pre-vaccination exposure to microbially rich and diverse soil and pneumococci-like bacteria elicits changes in IFN-γ and TGF-β secretion during pneumococcal vaccination. This indicates that skin exposure to microbially rich soil could have increased cell-mediated immunity to pneumococcal antigen. Although we report the association between soil pre-exposure and pneumonia vaccination, the response to other vaccines, such as influenza and Covid-19 vaccines, and various vaccine components, is a promising research lineage and deserves attention.

## Materials and methods

### Study group

Fifty healthy volunteers aged between 20 and 84 participated in the study (Table 1). Study subjects were recruited from November 2015 to April 2016. The interventions were administered between January and May in 2016, after which the trial ended. In the matched-pair design, participants were first matched in pairs according to similar gender, age, pet ownership and dwelling type (apartment building, terraced house, detached house). Then, within each pair, subjects were randomly assigned to the intervention group and to the control group (25 participants per group). Intended allocation ratio was 1:1. The randomization method was a simple randomization (mechanism: random number table) done by an independent researcher at University of Helsinki.

The medical exclusion criteria included immunosuppressive medications, immune deficiencies, at least infections within a year that resulted in hospitalization, a doctor-diagnosed memory disorder, a disease affecting immune response (e.g., colitis ulcerosa, rheumatoid arthritis, Crohn’s disease), Type 1 or Type 2 diabetes, acute earlier psychosis or acute depression, cancer diagnosis within the last 2 years or on-going cancer treatment, ulcers or rash in hands. Other exclusion criteria included daily smoking, incompetency and living outside city area. All participants provided a written, informed consent. Tetanus is a serious bacterial infection caused by the bacterium *Clostridium tetani* commonly found in soil. Therefore, protection against *Clostridium tetani* and immunological health status was confirmed before the trial using differential and complete blood count, and serum *C. tetani* tetanus toxoid antibodies from all study subjects at Fimlab Laboratories, Tampere, Finland, which is a certified hospital laboratory. If in any of the analyzed variables deviated from reference values, the study subject was excluded from the study. Just before the exposure period, a study nurse checked that participants’ hands were in good condition and the skin did not have wounds or eczema. There were no losses after randomization.

The study strictly followed the recommendations of the “*Finnish Advisory Board on Research Integrity*” and it received an approval from the ethics committee of the local hospital district (Pirkanmaa Hospital District, Finland). In accordance with the *Declaration of Helsinki*, written informed consents were received from all study participants.

The trial has been registered in ClinicalTrials.gov (ID NCT03351543) on 24/11/2017.

### Sample size

We used prior effect estimates from the studies that estimated immune function between people living in rich microbial environments and high hygiene environments^51–54^. These studies indicate that in Russian Karelia, where people are exposed to a greater number of microbes already in early childhood, the prevalence of type 1 diabetes, celiac disease, thyroid autoimmune diseases and allergic sensitization is 4–6 times lower than in Finland, even though the populations are genetically relatively close to each other. Based on this and our previous pilot study^20^, the microbial exposure causes an average of about 30% change in blood plasma cytokine levels, such as TGF-β, and in relative abundance of certain bacteria on the skin. When the significance level is set to p ≤ 0.001, difference between the means is 1.3, pooled standard deviation 1.14, and the statistical force is 0.8, the required number of pairs is 17. If the significance is set to p ≤ 0.01, the number of pairs required is 11. Power was calculated with paired t-tests of means in R environment: pwr.t.test(d = 1.3/1.14, power = 0.8,sig.level = 0.001,type = "paired", alternative = "greater"). As in one of our previous studies one third of study subjects cancelled the participation, 25 study subjects were recruited for each treatment group.

### Experimental design

**Figure 6:**
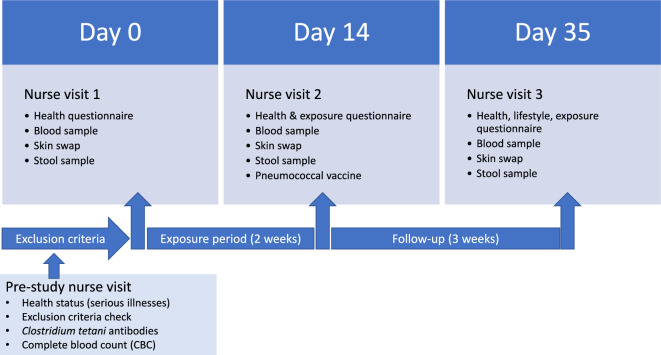
Study design. A study nurse interviewed the study participants and samples were taken before starting the exposure period (day 0), after the 2-week exposure period (day 14) and 3 weeks after the exposure period (day 35).

For the first 14 days, intervention group rubbed their hands with a soil- and plant-based material, three-times per day: before breakfast, before lunch and before dinner/evening snack. The 14-day exposure period was followed by a 3-week follow-up period (Fig. 6). The soil- and plant-based material has been described in detail in our previous studies^20,55^ and detailed metagenomic data can be found from Roslund et al.^40^. In short, the material contained composted ingredients comprising agricultural stack, gardening soils, deciduous leaf litter, peat, and *Sphangum* moss. Our pilot study has demonstrated that the material and the study method is safe and feasible^20^. The participants in the intervention treatment were instructed to rub soil material into their hands (for 20 s), after which hands were washed with tap water, without soap, for 5 s, and the hands were dabbed dry with a towel. On day 7, the material aliquot was replaced with new soil material, i.e., the aliquot. Control group did not receive any treatment, i.e. they continued their living habits as before the trial. Background information including medications, special diet, and living habits, were collected with questionnaires. All study participants were of normal weight.

#### Vaccination

All the study subjects received a pneumococcal vaccine (Prevenar 13, Pfizer) at the end of the exposure period (day 14) after giving blood samples, in order to test whether the immune response to vaccination is different between intervention and control group. Prevenar 13 is a conjugate vaccine with 13 different types of pneumococcal polysaccharide antigens (serotypes 1, 3, 4, 5, 6A, 7F, 9V, 14, 18C, 19A, 19F, 23F and 6B) conjugated to a carrier protein^30^. The vaccine was given as a single dose intramuscularly in the group muscle according to the manufacturer's instructions. The conjugate vaccine induces both T cell and B cell immunity.

### Sample collection

Blood and stool samples and skin swabs were collected at three time points; at baseline (day 0), after a two-week exposure period (day 14), and three weeks after the exposure period (day 35) (Fig. 1).

Skin swap samples were collected with a sterile cotton-wool stick wetted in 0.1% Tween 20 in 0.15 M NaCl from two parts of the dominant hand: (1) back of the hand (4 × 4 cm area), (2) forearm (5 × 5 cm area) (10 s wiping). The participants collected stool samples at home and they were stored in home freezers (− 18 to − 20 °C) for 1 to 2 days until they were transferred into − 70 °C.

The nurse took blood samples and interviewed the study participants about their health status on each visit. Nurse visits and data collection were at the Tampere University (Finland, Tampere) and at the University of Helsinki, Lahti campus (Finland, Lahti). In addition, adverse effects, potential lifestyle changes and adherence to the exposure protocol during the exposure period (e.g., nutrition, medication and dietary supplements) were recorded after the exposure period.

Samples were also collected from the intervention soil material before the exposure period and after the participants had rub their hands into it (day 7).

### Outcome measures

Primary measures were the differences in blood plasma TGF-β and other cytokine levels (IL-10, IL-17A, TNFα, and IFNγ) and in peripheral blood mononuclear cell (PBMC) cytokine production (IL-10, TNFα, IFNγ and TGF-β). To study PBMC cytokine production in detail, three separate stimulation treatments were performed. These were 1) autoclaved soil, 2) Prevenar 13 vaccine antigens and 3) CD3/CD28 antibody combination. A secondary outcome measure was to assess antibody response to Prevenar 13 vaccination, and if there are associations between cytokine levels and bacterial communities on the skin or in the gut. Secondary outcome measures included differences in bacterial diversity (beta and alpha), richness and relative abundance between intervention and control groups on skin and in the gut.

### DNA extraction, PCR and sequence processing

DNA was extracted with PowerSoil DNA Isolation Kit (MoBio Laboratories, Inc., Carlsbad, CA, USA) according to the manufacturer's standard protocol. The V4 region within the 16S rRNA gene was amplified by PCR (three technical replicates from each sample) using 505F and 806R primers^56^. Negative controls (sterile water) and a positive control (*Cupriavidus necator* JMP134, DSM 4058) was included in each PCR to ensure the quality of the analysis. Paired-end sequencing of the amplicons (2 X 300 bp) was performed on an Illumina Miseq instrument using a v3 reagent kit.

Raw paired-end sequence files were processed using Mothur version v1.35.1^57^ and as described earlier^4,45^. The forward and reverse sequence files were aligned into contigs and sequences that had any ambiguous bases, mismatch in the primers or DNA tag sequences, homopolymers longer than 8 bp, or an overlap shorter than 50 bp were removed. Sequences were aligned using the Mothur version of SILVA reference database v138^58^. Unique sequences that were almost identical (> 99%) were preclustered to remove erroneous reads^59^, and were screened for chimeras with UCHIME^60^. Non-chimeric sequences were assigned to taxa using the Naïve Bayesian Classifier^61^ against the RDP training set (version 10). Non-target sequences (mitochondria, chloroplast, Archaea) were removed. Sequences were clustered to amplicon sequence variants (ASVs) with 99% similarity using OptiClust. ASVs found in negative controls were removed from sequence data because of potential index hopping. If an ASV had an abundance ≤ 10 sequences across all the experimental units, the ASV was excluded from statistical analysis. The reason is that low-abundance OTUs typically are PCR or sequencing artifacts^62,63^. In addition, ASVs that were found only in one experimental unit were excluded from statistical analysis.

To conceptualize the different sequence read counts, zero count ASVs were replaced by an imputed value using the count zero multiplicative method from the zCompositions R package^64^ and samples were normalized with centered clr transformation^65–67^. Good's coverage index (average ± SD: soil 0.91 ± 0.06, stool 0.98 ± 0.01 and skin 0.92 ± 0.07) was used to determine ASV coverage adequacy for diversity and community composition analyses. We estimated richness and Shannon and Simpson diversity metrics for total bacterial communities in Mothur with the summary.single command. *Corynebacterium* ASVs were further identified with Nucleotide Basic Local Alignment Search Tool (BLASTN version 2.13.0).

### Separation of plasma and peripheral blood mononuclear cell (PBMC) samples

As described in Roslund et al. (2022)^18^, a venous blood sample was taken from the arm vein into Vacutainer CP Mononuclear Cell Preparation tubes with sodium citrate (BD Biosciences, NJ, USA). The samples were centrifuged following manufacturer's instructions to prepare the plasma and peripheral blood mononuclear cells (PBMCs). PBMCs were frozen in freezing medium consisting of 10% human AB serum (Sigma-Aldrich, MO, USA), 10% DMSO (Merck KGaA, Dgroupstadt, Germany), 50 µg/ml Streptomycin (Sigma-Aldrich, MO, USA) and 50 U/ml Penicillin, and 10 mM l-glutamine (Life Technologies, CA, USA) in RPMI-1640 medium (Life Technologies, CA, USA). Freezing containers at -80 °C (BioCision LLC, CA, USA) were used. The plasma samples were stored at -80 °C. The PBMC samples were transferred to -135 °C after 48h.

### PBMC stimulation

The cryopreserved PBMC samples were thawed, washed, and resuspended in complete RPMI-1640 medium (Life Technologies, CA, USA) supplemented with 50 U/ml Penicillin and 50 µg/ml Streptomycin (Sigma-Aldrich, MO, USA), 10% human AB serum (Sigma-Aldrich, MO, USA), and 10 mM l-glutamine (Life Technologies, CA, USA) at 1 × 106 cells/ml density. The cells were stimulated with Prevenar 13 vaccine antigens (0.5 µg/ml/antigen, except 1.0 µg/ml of serotype 6B antigen), anti-CD3 (5 μg/ml, BD Biosciences) and anti-CD28 (0.5 μg/ml, BD Biosciences) antibodies, autoclaved soil material filtered with a 35 µm filter and diluted to 1/100 or complete medium (negative control) at 37 °C 5% CO_2_. After 48 h incubation the supernatants were collected and stored at − 80 °C. Altogether, 566 PBMC stimulations were performed, including negative controls.

### Cytokine and antibody measurements

Cytokines were measured from both plasma and supernatant samples using the Milliplex MAP high sensitivity T cell panel kit (Merck KGaA, Dgroupstadt, Germany) and Human Cytokine/Chemokine Magnetic Bead Panel (Merck KGaA, Dgroupstadt, Germany) respectively. Fluorescence was analyzed using the Bio-Plex 200 system (Bio-Rad Laboratories, Hercules, CA, USA) and data were collected using the Bio-Plex Manager software (version 4.1, Bio-Rad Laboratories, Hercules, CA, USA). TGF-b concentrations were determined from plasma and supernatant samples by Human TGF-beta 1 ELISA kit (BioVendor, Czech Republic) and Human/Mouse TGF beta 1 Uncoated ELISA kit (Life Technologies, Carlsbad, CA, USA) respectively. For the plasma analysis kits the detection limits were IL-10: 0.56 pg/ml, IL-17A: 0.33 pg/ml, IFN-γ: 0.48 pg/ml, TNF-α: 0.16 pg/ml, and TGF-β: 6.0 pg/ml. For the supernatant analyses, the detection limits were IL-10: 1.1 pg/ml, IFN-γ: 0.8 pg/ml, TNF-α: 0.7 pg/ml, and TGF-β: 8 pg/ml. In the statistical analyses, the lowest detected concentration/2 (LOD/2) value was used in 3 IFN-γ samples because the cytokine concentration was below the limit of detection. Altogether, 150 plasma cytokine measurements were performed.

IgG class antibodies against Prevenar 13 vaccine antigens were measured by enzyme immunoassay (EIA) using the Prevenar 13 vaccine as antigen using similar EIA protocol as previously described^52,68,69^. Briefly, 96-well plates (Nunc Immuno plate, Maxisorb, Thermo Fisher Scientific, Waltham, MA, USA) were coated with 2.2 ng/well of each pneumococcal serotype (1, 3, 4, 5, 6A, 7F, 9V, 14, 18C, 19A, 19F, and 23F) antigen, except 4.4 ng/well of serotype 6B antigen. Plasma samples were analyzed in 1:2000 dilution in PBS supplemented with 1% bovine serum albumin, 2% NaCl and 0.05% Tween 20.

### Statistical analyses

All the statistical tests were done with R v3.6.1^70^*.* Linear mixed-effect models (LMM) [function *lmer* in *lme4* package^71^] were constructed to analyze temporal shifts in outcome measures, i.e., cytokines, pneumococcal antibodies and bacterial variables, taking into account pairing of intervention-control participants. Study subjects consuming probiotics during the trial were excluded from the cytokine, PBMC stimulation, antibody and gut microbiome analyses. In addition, study subjects using antibiotics during or max. 6 months before the trial were excluded from these statistical analsyses. The reason for these timeframes is that the effects of probiotics have been observed to disappear in a couple of days^72^, while the effects of antibiotics on gut microflora may persist for up to 6 months^73^. To estimate differences in cytokine changes between treatments, we used interaction between treatment arms and time in the LMM model, as recommended by Twisk et al.^74^. In detail, cytokine expression (plasma levels and in vitro stimulation measurements) was used as a dependent value, the interaction between treatment and time as an explanatory variable and paired participants as a grouping factor (Random factor) in LMM model. For each significant association, we used permutation tests (*lmperm* in *permuco* package) to run 5000 permutations to verify the P-value approximations^75^.

To test differences in bacterial measurements between treatments: bacterial richness, diversity or relative abundance was used as the dependent variable; treatment and time-point as a repeated measures factor (fixed factor); and paired participants as a grouping variable in LMM models. LMM models were performed for bacterial taxa with relative abundances of at least 0.1%. Permutation tests were used to verify the P-value approximations.

We calculated beta diversity of bacteria with Permutational Multivariate Analysis of Variance (PERMANOVA, function *adonis2* in *vegan* package) using Euclidean distances between clr transformed compositions^76^. We did two independent PERMANOVA analyses: one between time-points to estimate the changes within treatments and another within time-points to estimate between-treatment differences. PERMANOVA was performed at phylum, class, family, order, genus and ASV levels with abundance and presence/absence (standardization method “pa” with *decostand* function) datasets. Tests were carried out with Benjamini–Hochberg correction, to conceptualize the false discovery rate (FDR).

To test the correlation between bacterial community and cytokine expression, principal coordinates analysis (PCoA) with Euclidean distance was used to score the ASVs onto an ordination and correlation with corresponding cytokine expression levels and pneumococcal antibodies was assessed using function *envfit* in *vegan* package^77^ as in Roslund et al.^4^. Significance is tested by permutation test.

All the statistical tests were considered significant when permuted or FDR adjusted p-value was < 0.05 level.

### Supplementary Information

Supplementary Information.

## Data Availability

All bacterial sequence data were accessioned into the Sequence Read Archive (BioProject ID: PRJNA881933). All other data needed to support the conclusions of this manuscript are included in the main text and supplementary appendix. The sensitive data that support the findings of this study are available from University of Helsinki but restrictions defined in General Data Protection Regulation (EU 2016/679) and Finnish Data Protection Act 1050/2018 apply to the availability of these data, and so are not publicly available. Data are however available from the authors upon reasonable request and with permission from the ethical committee of the local hospital district (Tampereen yliopistollisen sairaalan erityisvastuualue, Pirkanmaa, Finland).
