# Supplementary material for: Skin exposure to soil microbiota elicits changes in cell-mediated immunity to pneumococcal vaccine
Source: Sci Rep. 2024 Aug 10;14:18573. doi: 10.1038/s41598-024-68235-8 (PMC11316737; doi:10.1038/s41598-024-68235-8)
Supplement: Supplementary file 1 — Supplementary Information. [file 41598_2024_68235_MOESM1_ESM.pdf]

Supplementary Materials For

Skin exposure to soil microbiota elicits changes in cell-mediated immunity to pneumococcal vaccine

Marja I. Roslund[1], Noora Nurminen[2], Sami Oikarinen[2], Riikka Puhakka[3], Mira Grönroos[3], Leena Puustinen[2], Laura Kummola[2], Anirudra Parajuli[3‡], Ondřej Cinek[4], Olli H. Laitinen[2], Heikki Hyöty[2,5], Aki Sinkkonen[1*]

[1]Natural Resources Institute Finland, Luke, Viikki and Turku, Finland

[2]Faculty of Medicine and Health Technology, Tampere University, Arvo Ylpön katu 34, FI-33520 Tampere, Finland

[3]Ecosystems and Environment Research Programme, Faculty of Biological and Environmental Sciences, University of Helsinki, Niemenkatu 73, FI-15140 Lahti, Finland

[4]Department of Medical Microbiology, 2nd Faculty of Medicine, Charles University, V Úvalu 84, Praha 5, 150 06 Prague, Czech Republic

[5]Fimlab Laboratories, Pirkanmaa Hospital District, Tampere, Finland

‡The current affiliation for Parajuli A. is Department of Medicine, Karolinska Institutet, Huddinge, Sweden

*Corresponding author: aki.sinkkonen@luke.fi

The PDF file includes
Tables S1 to S6
Figs. S1 and S2

Table S1. A) Characteristics of study participants included in cytokine measurements (n=15 in intervention and control treatments). Age is presented as mean ± standard deviation. Outdoor recreations are presented at nominal scale (median ± confidence level 95% = cl): 1 = not at all, 2 = rarely, 3 = monthly, and 4 = weekly. B) Principal component analysis (PcOA) for living habits were done with envfit function in R environment. PcOA statistics are reported as R squared (R2) and probapility p value. C) Concentrations of all cytokines measured in plasma and peripheral blood mononuclear cells (PBMCs) stimulated with prevenar vaccine antigens, autoclaved soil and CD3/CD28 antigens at each time point (day 0, day 14 and day 35).

**A) Charasteristics of study participants**	**Intervention**	**Control**	**Total**
Gender, male	3	4	7
Gender, female	12	11	23
Gender, other	0	0	0
Age	55 ± 19	62 ± 17	59 ± 18
Dwelling type:			
Detached house	1	2	3
Apartment building	14	13	27
Terraced house	0	0	0
Pet ownership	3	3	6
Outdoor recreation:			
Gardening	2 ± 0.61	2 ± 0.61	2 ± 0.44
Walking	4 ± 0.48	4 ± 0.39	4 ± 0.31
Cycling	2 ± 0.70	4 ± 0.64	4 ± 0.48
Hiking	1.5 ± 0.47	2 ± 0.47	2 ± 0.34
Berrypicking	2 ± 0.48	3 ± 0.51	2 ± 0.36
Mushroompicking	2 ± 0.53	2 ± 0.58	2 ± 0.39
Hunting	1 ± 0.43	1 ± 0	1 ± 0.21
Fishing	1 ± 0.44	2 ± 0.39	2 ± 0.30
Birdwatching	2 ± 0.63	2 ± 0.50	2 ± 0.41

**B) Principal component analysis for living habits**		
	**R2**	**p value**
Pet ownership	0.0218	0.671
Gardening	0.0311	0.903
Walking	0.152	0.082
Cycling	0.1176	0.68
Hiking	0.2579	0.219
Berrypicking	0.2142	0.318
Mushroompicking	0.1154	0.669
Hunting	0.1505	0.305
Fishing	0.1168	0.693
Birdwatching	0.2107	0.134

C) Concentrations of all cytokines measured at each time point.

	Intervention					
	Day 0		Day 14		Day 35	
**Plasma cytokines**	**Mean**	**sd**	**Mean**	**sd**	**Mean**	**sd**
IFN-γ (pg ml-1)	20.70	12.96	18.34	11.42	22.76	16.10
IL-10 (pg ml-1)	21.99	12.17	19.66	14.32	23.75	19.74
TNF-α (pg ml-1)	2.81	0.97	2.72	1.37	3.06	1.77
TGF-β (ng ml-1)	16.11	7.63	16.16	9.32	12.57	7.73
IL-17A (pg ml-1)	6.89	3.09	6.40	3.72	7.79	5.31
**PBMC stimulation: Prevenar**	**Mean**	**sd**	**Mean**	**sd**	**Mean**	**sd**
IFN-γ (pg ml-1)	6.44	10.82	38.89	67.08	17.31	35.59
IL-10 (pg ml-1)	82.93	82.70	75.96	72.60	73.62	49.25
TNF-α (pg ml-1)	339.93	334.51	371.99	546.81	312.64	241.27
TGF-β (pg ml-1)	1003.02	369.52	992.54	348.68	957.45	202.72
**PBMC stimulation: Autoclaved soil**	**Mean**	**sd**	**Mean**	**sd**	**Mean**	**sd**
IFN-γ (pg ml-1)	64.13	122.98	77.78	105.44	42.44	48.10
IL-10 (pg ml-1)	923.09	1128.82	980.94	1697.10	665.56	509.02
TNF-α (pg ml-1)	3142.38	7705.50	788.94	841.06	671.52	451.33
TGF-β (pg ml-1)	834.74	356.45	963.91	299.83	956.91	349.49
**PBMC stimulation: CD3-CD28**	**Mean**	**sd**	**Mean**	**sd**	**Mean**	**sd**
IFN-γ (pg ml-1)	4323.12	6209.56	3980.13	4937.52	6567.93	8429.92
IL-10 (pg ml-1)	1364.57	2430.53	964.72	1046.20	1335.19	1320.35
TNF-α (pg ml-1)	828.58	632.96	641.07	384.40	904.61	730.79
TGF-β (pg ml-1)	983.25	390.69	953.81	323.46	984.43	285.34

	Control					
	Day 0		Day 14		Day 35	
**Plasma cytokines**	**Mean**	**sd**	**Mean**	**sd**	**Mean**	**sd**
IFN-γ (pg ml-1)	21.49	11.65	20.56	11.94	21.71	15.99
IL-10 (pg ml-1)	23.90	12.34	22.10	13.01	24.04	18.32
TNF-α (pg ml-1)	3.18	1.02	2.73	1.68	2.65	1.55
TGF-β (ng ml-1)	20.65	9.44	16.61	9.24	11.53	8.99
IL-17A (pg ml-1)	7.89	3.81	7.26	6.09	7.72	6.01
**PBMC stimulation: Prevenar**	**Mean**	**sd**	**Mean**	**sd**	**Mean**	**sd**
IFN-γ (pg ml-1)	6.80	8.39	3.91	3.60	30.61	41.99
IL-10 (pg ml-1)	93.51	79.73	103.68	118.57	109.29	65.20
TNF-α (pg ml-1)	364.28	320.67	336.58	317.11	286.15	165.66
TGF-β (pg ml-1)	957.65	371.99	860.81	410.47	1029.29	217.31
**PBMC stimulation: Autoclaved soil**	**Mean**	**sd**	**Mean**	**sd**	**Mean**	**sd**
IFN-γ (pg ml-1)	60.23	71.88	22.52	22.23	45.68	46.36
IL-10 (pg ml-1)	878.03	583.91	858.19	523.70	881.10	492.96
TNF-α (pg ml-1)	759.47	311.95	699.87	509.65	559.79	378.60
TGF-β (pg ml-1)	903.09	498.98	919.12	266.46	959.74	428.67
**PBMC stimulation: CD3-CD28**	**Mean**	**sd**	**Mean**	**sd**	**Mean**	**sd**
IFN-γ (pg ml-1)	5185.74	6888.41	4152.31	5394.99	5306.64	7978.97
IL-10 (pg ml-1)	1094.16	1581.08	862.31	829.33	1274.81	2049.13
TNF-α (pg ml-1)	773.57	682.29	681.68	617.33	742.71	679.12
TGF-β (pg ml-1)	859.19	393.07	874.20	336.57	829.00	277.89

Table S2. Linear mixed model (LMM) results for plasma cytokines (pg ml-1) and pneumococcal antibodies (EIU ml -1) A) between treatmen groups, and within B) intervention and C) control treatment groups. PBMCs were stimulated with Prevenar 13 vaccine antigens, autoclaved soil material, and anti-CD3 and anti-CD28, and within control group between intervention and control treatment groups. Data is presented as mean ± standard deviation (sd). LMM statistics are reported as t value, probability P value, and R squared (R2) for fixed factor.

**A) Within intervention treatment group**	**Day 0**		**Day 14**		**Day 35**	
	**Mean**	**sd**	**Mean**	**sd**	**Mean**	**sd**
**IL-10 (pg ml-1)**	21.995	12.172	19.657	14.321	23.753	19.742
**IL-17A (pg ml-1)**	6.894	3.094	6.395	3.718	7.788	5.311
**TNF-α (pg ml-1)**	2.807	0.965	2.717	1.373	3.063	1.773
**IFN-γ (pg ml-1)**	20.699	12.964	18.343	11.425	22.759	16.100
**TGF-β (ng ml-1)**	16.112	7.628	16.155	9.317	12.570	7.726
**Pneumococcal antibodies**	11.200	12.648	11.978	14.330	115.445	61.798

	**LMM: Day 0 vs Day 14**		**LMM: Day 0 vs Day 35**			**R squared**
**LMM statistics:**	**t value**	**p value**	**t value**	**p value**	**perm.p**	**Fixed R2**
**IL-10 (pg ml-1)**	-0.527	0.598	0.396	0.692		-0.058
**IL-17A (pg ml-1)**	-0.413	0.679	0.741	0.459		-0.075
**TNF-α (pg ml-1)**	-0.203	0.839	0.577	0.564		-0.068
**IFN-γ (pg ml-1)**	-0.642	0.521	0.561	0.575		-0.058
**TGF-β (ng ml-1)**	0.028	0.978	-2.280	**0.023**	0.069	0.036
**Pneumococcal antibodies**	0.061	0.951	8.166	**0.000**	**0.000**	0.636

**B) Within control treatment group**	**Day 0**		**Day 14**		**Day 35**	
	**Mean**	**sd**	**Mean**	**sd**	**Mean**	**sd**
**IL-10 (pg ml-1)**	23.903	12.339	22.100	13.012	24.035	18.320
**IL-17A (pg ml-1)**	7.887	3.809	7.256	6.089	7.719	6.014
**TNF-α (pg ml-1)**	3.184	1.023	2.734	1.679	2.654	1.554
**IFN-γ (pg ml-1)**	21.489	11.652	20.559	11.941	21.709	15.987
**TGF-β (ng ml-1)**	20.645	9.439	16.610	9.242	11.534	8.990
**Pneumococcal antibodies**	12.726	14.051	13.834	15.455	126.226	74.212

**LMM statistics:**	**Day 0 vs Day 14**		**Day 0 vs Day 35**			**R squared**
	**t value**	**p value**	**t value**	**p value**	**perm.p**	**Fixed R2**
**IL-10 (pg ml-1)**	-0.467	0.641	-0.072	0.943		0.002
**IL-17A (pg ml-1)**	-0.005	0.996	-0.287	0.774		-0.001
**TNF-α (pg ml-1)**	-1.059	0.289	-1.613	0.107		0.026
**IFN-γ (pg ml-1)**	-0.218	0.827	-0.065	0.948		0.001
**TGF-β (ng ml-1)**	-1.588	0.112	-5.207	**0.000**	**0.000**	0.143
**Pneumococcal antibodies**	0.255	0.799	8.215	**0.000**	**0.000**	0.609

C) Change difference between treatments LMM statistics:	Day 0 vs Day 14 t value	p value	Day 0 vs Day 35 t value	p value	perm.p	R squared Fixed R2
**IL-10 (pg ml-1)**	-0.143	0.886	0.358	0.721		-0.001
**IL-17A (pg ml-1)**	-0.284	0.776	0.724	0.469		0.004
**TNF-α (pg ml-1)**	0.468	0.640	1.364	0.173		0.009
**IFN-γ (pg ml-1)**	-0.382	0.703	0.498	0.619		-0.012
**TGF-β (ng ml-1)**	1.184	0.236	2.350	**0.019**	**0.024**	0.101
**Pneumococcal antibodies**	-0.044	0.965	-0.498	0.619		0.627

LMM statistics:	Day 14 vs Day 35 t value	p value	R squared Fixed R2
**IL-10 (pg ml-1)**	0.625	0.532	0.001
**IL-17A (pg ml-1)**	1.749	0.080	-0.001
**TNF-α (pg ml-1)**	1.206	0.228	-0.003
**IFN-γ (pg ml-1)**	1.058	0.290	0.000
**TGF-β (ng ml-1)**	1.264	0.206	0.043
**Pneumococcal antibodies**	-0.376	0.707	0.565

Table S3. Linear mixed model (LMM) results for PBMC stimulation (pg ml-1) A) between treatmen groups, and within B) intervention and C) control treatment groups. PBMCs were stimulated with Prevenar 13 vaccine antigens, autoclaved soil material, and anti-CD3 and anti-CD28. Data is presented as mean ± standard deviation (sd). LMM statistics are reported as t value, probability P value, and R squared (R2) for fixed factor, and random variance for random factors. Permutation test with 5,000 permutations was used to verify the p value approximations (perm. p).

**A) Change difference between treatment groups**	**LMM statistics Day 0 vs Day 14**			**LMM statistics Day 0 vs Day 35**		**R squared**
**Prevenar 13 vaccine antigen**	**t value**	**p value**	**Perm. p**	**t value**	**p value**	**Fixed R2**
**IFN-γ**	2.495	**0.013**	**0.048**	-0.610	0.542	0.169
**IL-10**	-0.338	0.736		-0.993	0.321	0.030
**TNF-α**	0.537	0.591		-0.350	0.726	-0.002
**TGF-β**	0.921	0.357		-0.641	0.522	0.050
	**Day 0 vs Day 14**			**Day 0 vs Day 35**		**R squared**
**Autoclaved soil material**	**t value**	**p value**		**t value**	**p value**	**Fixed R2**
**IFN-γ**	0.895	0.371		-0.100	0.921	0.011
**IL-10**	0.419	0.675		0.143	0.886	-0.018
**TNF-α**	-0.895	0.371		-0.927	0.354	0.085
**TGF-β**	0.800	0.424		1.245	0.213	0.043
	**Day 0 vs Day 14**			**Day 0 vs Day 35**		**R squared**
**Anti CD3-CD28**	**t value**	**p value**		**t value**	**p value**	**Fixed R2**
**IFN-γ**	0.665	0.506		0.778	0.436	0.008
**IL-10**	-0.404	0.686		-0.305	0.761	0.013
**TNF-α**	-0.545	0.586		0.120	0.904	0.020
**TGF-β**	-0.282	0.778		0.295	0.768	0.032

	**LMM statistics Day 14 vs Day 35**			**R squared**	**Random variance**	
**Prevenar 13 vaccine antigen**	**t value**	**p value**	**Perm. p**	**Fixed R2**	**Age**	**Sex**
**IFN-γ**	-2.778	**0.005**	**0.037**	0.080	0	0
**IL-10**	-0.361	0.718		0.047	1597	0
**TNF-α**	-0.413	0.680		-0.001	75156	0
**TGF-β**	-1.347	0.178		0.056	0	0
	**LMM statistics Day 14 vs Day 35**			**R squared**	**Random variance**	
**Autoclaved soil material**	**t value**	**p value**		**Fixed R2**	**Age**	**Sex**
**IFN-γ**	-0.993	0.321		0.052	2834	0
**IL-10**	0.151	0.880		-0.026	0	0
**TNF-α**	-0.029	0.977		0.069	1027363	276282
**TGF-β**	1.094	0.274		0.065	0	0
	**LMM statistics Day 14 vs Day 35**			**R squared**	**Random variance**	
**Anti CD3-CD28**	**t value**	**p value**		**Fixed R2**	**Age**	**Sex**
**IFN-γ**	0.333	0.739		0.023	0	0
**IL-10**	0.686	0.492		0.010	0	0
**TNF-α**	1.040	0.298		0.024	0	0
**TGF-β**	0.620	0.535		0.036	0	16583

B) Within intervention treatment group<br>Prevenar 13 vaccine antigen	Day 0<br>Mean	sd	Day 14<br>Mean	sd	Day 35<br>Mean	sd
IFN-γ	6	11	39	67	17	36
IL-10	83	83	76	73	74	49
TNF-α	340	335	372	547	313	241
TGF-β	1003	370	993	349	957	203
**Autoclaved soil material**	**Day 0<br>Mean**	**sd**	**Day 14<br>Mean**	**sd**	**Day 35<br>Mean**	**sd**
IFN-γ	64	123	78	105	42	48
IL-10	923	1129	981	1697	666	509
TNF-α	3142	7706	789	841	672	451
TGF-β	835	356	964	300	957	349
**Anti CD3-CD28**	**Day 0<br>Mean**	**sd**	**Day 14<br>Mean**	**sd**	**Day 35<br>Mean**	**sd**
IFN-γ	4323	6210	3980	4938	6568	8430
IL-10	1365	2431	965	1046	1335	1320
TNF-α	829	633	641	384	905	731
TGF-β	983	391	954	323	984	285

Prevenar 13 vaccine antigen	LMM statistics<br>Day 0 vs Day 14<br>t value	p value	Perm. p	LMM statistics<br>Day 0 vs Day 35<br>t value	p value	R squared<br>fixed.R2
IFN-γ	2.550	**0.011**	**0.024**	1.031	0.302	0.086
IL-10	-0.078	0.938		-0.536	0.592	0.001
TNF-α	0.356	0.722		-0.318	0.751	0.004
TGF-β	-0.135	0.892		-0.393	0.694	0.004
**Autoclaved soil material**	**t value**	**p value**		**t value**	**p value**	**fixed.R2**
IFN-γ	0.432	0.666		-0.314	0.754	0.011
IL-10	0.217	0.828		0.346	0.729	-0.012
TNF-α	-1.183	0.237		-1.236	0.217	0.066
TGF-β	1.172	0.241		1.647	0.100	0.076
**Anti CD3-CD28**	**t value**	**p value**	**Perm. p**	**t value**	**p value**	**fixed.R2**
IFN-γ	-0.260	0.795		1.609	0.108	0.030
IL-10	-0.854	0.393		-0.082	0.935	0.012
TNF-α	-2.055	**0.040**	**0.035**	0.721	0.471	0.035
TGF-β	-0.327	0.744		-0.071	0.944	0.002

C) Within control treatment group<br>Prevenar 13 vaccine antigen	Day 0<br>Mean	sd	Day 14<br>Mean	sd	Day 35<br>Mean	sd
IFN-γ	7	8	4	4	31	42
IL-10	94	80	104	119	109	65
TNF-α	364	321	337	317	286	166
TGF-β	958	372	861	410	1029	217
**Autoclaved soil material**	**Day 0**<br>**Mean**	**sd**	**Day 14**<br>**Mean**	**sd**	**Day 35**<br>**Mean**	**sd**
IFN-γ	60	72	23	22	46	46
IL-10	878	584	858	524	881	493
TNF-α	759	312	700	510	560	379
TGF-β	903	499	919	266	960	429
**Anti CD3-CD28**	**Day 0**<br>**Mean**	**sd**	**Day 14**<br>**Mean**	**sd**	**Day 35**<br>**Mean**	**sd**
IFN-γ	5186	6888	4152	5395	5307	7979
IL-10	1094	1581	862	829	1275	2049
TNF-α	774	682	682	617	743	679
TGF-β	859	393	874	337	829	278

Prevenar 13 vaccine antigen	LMM Day 0 vs Day 14<br>t value	p value	LMM Day 0 vs Day 35<br>t value	p value	Perm. p	R squared<br>fixed.R2
IFN-γ	-0.326	0.745	2.792	**0.005**	**0.003**	0.222
IL-10	0.328	0.743	0.656	0.512		0.009
TNF-α	-0.604	0.546	-0.579	0.562		0.006
TGF-β	-0.981	0.327	0.490	0.624		0.039
**Autoclaved soil material**	**LMM Day 0 vs Day 14**<br>**t value**	**p value**	**LMM Day 0 vs Day 35**<br>**t value**	**p value**	**Perm. p**	**R squared**<br>**fixed.R2**
IFN-γ	-1.065	0.287	-0.162	0.871		0.064
IL-10	-0.530	0.596	0.182	0.856		0.011
TNF-α	-1.922	0.055	-3.157	**0.002**	**0.001**	0.314
TGF-β	-0.142	0.887	-0.257	0.797		0.003
**Anti CD3-CD28**	**LMM Day 0 vs Day 14**<br>**t value**	**p value**	**LMM Day 0 vs Day 35**<br>**t value**	**p value**	**Perm. p**	**R squared**<br>**fixed.R2**
IFN-γ	-1.157	0.247	0.499	0.618		0.001
IL-10	-0.243	0.808	0.391	0.696		0.011
TNF-α	-0.582	0.561	0.243	0.808		0.002
TGF-β	0.116	0.907	-0.278	0.781		0.003

Table S4. Permutational multivariate analysis of variance (PERMANOVA) results for skin bacterial communities on the back of the hand. Results are reported on day 0 (A), day 14 (B) and day 35 (C) between intervention and control treatment groups, and within intervention (D) and control (E) treatments. Statistics are shown as F value, R squared, probability P value and Benjamini–Hochberg adjusted Q value for abundance (weighted) data.

**A) Day 0: Intervention vs. control**	**F value**	**R2**	**p value**	**Q value**
Total bacterial communtiy (ASV level)	0.74	0.02	0.968	0.999
Phyla Firmicutes	0.80	0.02	0.883	0.999
Phyla Proteobacteria	0.69	0.02	0.984	0.999
Phyla Actinobacteria	0.76	0.02	0.939	0.999
Phyla Bacteroidetes	0.59	0.02	0.994	0.999
Phyla Verrucomicrobia	0.86	0.02	0.658	0.999
Phyla Fusobacteria	0.63	0.02	0.885	0.999
Class Bacteroidia	0.52	0.02	0.999	0.999
Class Clostridia	0.82	0.02	0.831	0.999
Class Negativicutes	0.90	0.03	0.615	0.999
Class Bacilli	0.80	0.02	0.872	0.999
Class Sphingobacteriia	0.77	0.02	0.655	0.999
Class Flavobacteriia	0.60	0.02	0.933	0.999
Class Gammaproteobacteria	0.70	0.02	0.985	0.999
Class Alphaproteobacteria	0.71	0.02	0.929	0.999
Order Lactobacillales	0.75	0.03	0.915	0.999
Family Thermoactinomycetaceae1	0.95	0.03	0.380	0.999
Genus Staphylococcus	0.88	0.03	0.673	0.999
Genus Streptococcus	0.85	0.02	0.705	0.999
Alphaproteobacteria unclassified genus	0.68	0.02	0.730	0.999
**B) Day 14: Intervention vs. control**	**F value**	**R2**	**p value**	**Q value**
Total bacterial communtiy (ASV level)	1.66	0.05	**0.002**	**0.012**
Phyla Firmicutes	1.64	0.05	**0.005**	**0.010**
Phyla Proteobacteria	1.75	0.05	**0.004**	**0.010**
Phyla Actinobacteria	1.61	0.05	**0.002**	**0.010**
Phyla Bacteroidetes	1.70	0.05	**0.004**	**0.010**
Phyla Verrucomicrobia	1.51	0.04	**0.028**	0.050
Phyla Fusobacteria	1.10	0.03	0.339	0.424
Class Bacteroidia	1.64	0.05	**0.003**	**0.008**
Class Clostridia	1.71	0.05	**0.003**	**0.008**
Class Negativicutes	2.29	0.06	**0.001**	**0.006**
Class Bacilli	1.52	0.04	**0.010**	**0.019**
Class Sphingobacteriia	1.25	0.04	0.246	0.312
Class Flavobacteriia	1.83	0.05	**0.004**	**0.010**
Class Gammaproteobacteria	1.71	0.05	**0.005**	**0.011**
Class Alphaproteobacteria	1.92	0.05	**0.003**	**0.008**
Order Lactobacillales	1.75	0.06	**0.001**	**0.006**
Family Thermoactinomycetaceae1	4.75	0.12	**0.018**	**0.029**
Genus Staphylococcus	1.54	0.04	**0.011**	**0.029**
Genus Streptococcus	1.76	0.05	**0.007**	**0.029**
Alphaproteobacteria unclassified genus	1.17	0.03	0.285	0.312

**C) Day 35: Intervention vs. control**	**F value**	**R2**	**p value**	**Q value**
Total bacterial communtiy (ASV level)	0.96	0.03	0.470	0.753
Phyla Firmicutes	0.92	0.03	0.617	0.808
Phyla Proteobacteria	1.00	0.03	0.376	0.752
Phyla Actinobacteria	0.96	0.03	0.475	0.792
Phyla Bacteroidetes	1.05	0.03	0.311	0.752
Phyla Verrucomicrobia	1.58	0.04	**0.017**	0.102
Phyla Fusobacteria	0.77	0.02	0.727	0.808
Class Bacteroidia	0.99	0.03	0.420	0.626
Class Clostridia	0.87	0.03	0.684	0.722
Class Negativicutes	1.00	0.03	0.428	0.626
Class Bacilli	0.93	0.03	0.566	0.633
Class Sphingobacteriia	1.15	0.03	0.290	0.561
Class Flavobacteriia	0.97	0.03	0.505	0.633
Class Gammaproteobacteria	0.93	0.03	0.566	0.633
Class Alphaproteobacteria	1.15	0.03	0.255	0.561
Order Lactobacillales	1.01	0.04	0.384	0.561
Family Thermoactinomycetaceae1	3.01	0.08	**0.043**	0.151
Genus Staphylococcus	0.90	0.03	0.591	0.726
Genus Streptococcus	0.92	0.03	0.622	0.726
Alphaproteobacteria unclassified genus	2.66	0.07	**0.007**	**0.049**
**D) Intervention**				
**Day 0 vs 14**	**F value**	**R2**	**p value**	**Q value**
Total bacterial communtiy (ASV level)	1.92	0.05	**0.004**	**0.009**
Phyla Firmicutes	1.93	0.05	**0.002**	**0.006**
Phyla Proteobacteria	1.90	0.05	**0.002**	**0.006**
Phyla Actinobacteria	1.89	0.05	**0.001**	**0.005**
Phyla Bacteroidetes	1.95	0.05	**0.001**	**0.005**
Phyla Verrucomicrobia	2.38	0.07	**0.002**	**0.006**
Phyla Fusobacteria	1.54	0.04	0.074	0.094
Class Bacteroidia	1.90	0.05	**0.001**	**0.005**
Class Clostridia	2.13	0.06	**0.001**	**0.005**
Class Negativicutes	2.52	0.07	**0.001**	**0.005**
Class Bacilli	1.79	0.05	**0.003**	**0.008**
Class Sphingobacteriia	1.42	0.04	0.160	0.177
Class Flavobacteriia	1.86	0.05	**0.003**	**0.008**
Class Gammaproteobacteria	1.90	0.05	**0.006**	**0.011**
Class Alphaproteobacteria	1.86	0.05	**0.005**	**0.009**
Order Lactobacillales	1.98	0.07	**0.001**	**0.005**
Family Thermoactinomycetaceae1	7.00	0.17	**0.005**	**0.009**
Genus Staphylococcus	1.84	0.05	**0.002**	**0.006**
Genus Streptococcus	1.92	0.05	**0.010**	**0.016**
Alphaproteobacteria unclassified genus	1.98	0.07	**0.001**	**0.005**

**D) Intervention**				
**Day 0 vs 35**	**F value**	**R2**	**p value**	**Q value**
Total bacterial communtiy (ASV level)	0.88	0.03	0.884	0.884
Phyla Firmicutes	0.91	0.03	0.787	0.992
Phyla Proteobacteria	0.84	0.02	0.954	0.992
Phyla Actinobacteria	0.91	0.03	0.810	0.992
Phyla Bacteroidetes	0.93	0.03	0.706	0.992
Phyla Verrucomicrobia	0.57	0.02	0.992	0.992
Phyla Fusobacteria	0.81	0.02	0.741	0.992
Class Bacteroidia	0.98	0.03	0.502	0.986
Class Clostridia	0.91	0.03	0.729	0.986
Class Negativicutes	0.96	0.03	0.531	0.986
Class Bacilli	0.92	0.03	0.733	0.986
Class Sphingobacteriia	0.77	0.02	0.731	0.986
Class Flavobacteriia	0.79	0.02	0.817	0.986
Class Gammaproteobacteria	0.85	0.02	0.903	0.986
Class Alphaproteobacteria	0.81	0.02	0.871	0.986
Order Lactobacillales	1.17	0.04	0.191	0.191
Family Thermoactinomycetaceae1	2.41	0.07	**0.039**	0.078
Genus Staphylococcus	0.82	0.02	0.868	0.980
Genus Streptococcus	1.19	0.03	0.132	0.453
Alphaproteobacteria unclassified genus	1.28	0.04	0.194	0.453
**Day 14 vs 35**	**F value**	**R2**	**p value**	**Q value**
Total bacterial communtiy (ASV level)	1.37	0.04	**0.034**	**0.041**
Phyla Firmicutes	1.36	0.04	**0.039**	0.098
Phyla Proteobacteria	1.40	0.04	**0.038**	0.098
Phyla Actinobacteria	1.36	0.04	**0.049**	0.098
Phyla Bacteroidetes	1.39	0.04	**0.044**	0.098
Phyla Verrucomicrobia	1.75	0.05	**0.040**	0.098
Phyla Fusobacteria	1.30	0.04	0.161	0.201
Class Bacteroidia	1.32	0.04	0.053	0.110
Class Clostridia	1.50	0.04	**0.021**	0.103
Class Negativicutes	1.95	0.05	**0.004**	0.076
Class Bacilli	1.24	0.04	0.092	0.159
Class Sphingobacteriia	0.91	0.03	0.563	0.608
Class Flavobacteriia	1.73	0.05	**0.012**	0.103
Class Gammaproteobacteria	1.33	0.04	0.058	0.110
Class Alphaproteobacteria	1.54	0.04	**0.039**	0.106
Order Lactobacillales	1.25	0.05	0.085	0.100
Family Thermoactinomycetaceae1	1.08	0.03	0.311	0.364
Genus Staphylococcus	1.16	0.03	0.217	0.364
Genus Streptococcus	1.69	0.05	**0.017**	0.119
Alphaproteobacteria unclassified genus	1.40	0.04	0.182	0.364

**E) Control**				
**Day 0 vs 14**	**F value**	**R2**	**p value**	**Q value**
Total bacterial communtiy (ASV level)	1.29	0.04	0.082	0.220
Phyla Firmicutes	1.37	0.04	**0.042**	0.206
Phyla Proteobacteria	1.26	0.04	0.104	0.243
Phyla Actinobacteria	1.25	0.04	0.119	0.256
Phyla Bacteroidetes	1.33	0.04	0.073	0.219
Phyla Verrucomicrobia	1.03	0.03	0.429	0.594
Phyla Fusobacteria	1.47	0.04	0.122	0.256
Class Bacteroidia	1.40	0.04	0.059	0.219
Class Clostridia	1.41	0.04	**0.047**	0.206
Class Negativicutes	1.49	0.04	**0.042**	0.206
Class Bacilli	1.37	0.04	**0.049**	0.206
Class Sphingobacteriia	1.36	0.04	0.200	0.336
Class Flavobacteriia	1.43	0.04	0.087	0.220
Class Gammaproteobacteria	1.23	0.03	0.132	0.264
Class Alphaproteobacteria	1.34	0.04	0.071	0.219
Order Lactobacillales	1.25	0.05	0.153	0.336
Family Thermoactinomycetaceae1	0.81	0.02	0.467	0.594
Genus Staphylococcus	1.07	0.03	0.307	0.478
Genus Streptococcus	1.72	0.05	**0.016**	0.206
Alphaproteobacteria unclassified genus	1.39	0.04	0.199	0.336
**Day 0 vs 35**	**F value**	**R2**	**p value**	**Q value**
Total bacterial communtiy (ASV level)	0.73	0.02	0.965	0.999
Phyla Firmicutes	0.77	0.02	0.890	0.999
Phyla Proteobacteria	0.68	0.02	0.982	0.999
Phyla Actinobacteria	0.66	0.02	0.979	0.999
Phyla Bacteroidetes	0.88	0.03	0.662	0.999
Phyla Verrucomicrobia	1.52	0.04	0.068	0.999
Phyla Fusobacteria	0.65	0.02	0.812	0.999
Class Bacteroidia	0.91	0.03	0.582	0.999
Class Clostridia	0.82	0.02	0.751	0.999
Class Negativicutes	0.46	0.01	0.998	0.999
Class Bacilli	0.75	0.02	0.926	0.999
Class Sphingobacteriia	0.95	0.03	0.471	0.999
Class Flavobacteriia	0.88	0.03	0.601	0.999
Class Gammaproteobacteria	0.65	0.02	0.987	0.999
Class Alphaproteobacteria	0.84	0.02	0.739	0.999
Order Lactobacillales	0.70	0.03	0.919	0.999
Family Thermoactinomycetaceae1	0.63	0.02	0.517	0.999
Genus Staphylococcus	0.52	0.02	0.999	0.999
Genus Streptococcus	0.84	0.02	0.709	0.999
Alphaproteobacteria unclassified genus	1.23	0.03	0.256	0.999

**E) Control**				
**Day 14 vs 35**	**F value**	**R2**	**p value**	**Q value**
Total bacterial communtiy (ASV level)	1.18	0.03	0.149	0.618
Phyla Firmicutes	1.22	0.03	0.110	0.247
Phyla Proteobacteria	1.18	0.03	0.148	0.247
Phyla Actinobacteria	1.12	0.03	0.222	0.317
Phyla Bacteroidetes	1.21	0.03	0.129	0.247
Phyla Verrucomicrobia	1.98	0.06	**0.005**	**0.050**
Phyla Fusobacteria	1.49	0.04	0.107	0.247
Class Bacteroidia	1.32	0.04	0.090	0.354
Class Clostridia	1.27	0.04	0.092	0.354
Class Negativicutes	1.01	0.03	0.440	0.569
Class Bacilli	1.22	0.03	0.149	0.354
Class Sphingobacteriia	1.27	0.04	0.244	0.500
Class Flavobacteriia	1.00	0.03	0.449	0.569
Class Gammaproteobacteria	1.09	0.03	0.263	0.500
Class Alphaproteobacteria	1.41	0.04	0.062	0.354
Order Lactobacillales	1.14	0.04	0.246	0.700
Family Thermoactinomycetaceae1	0.81	0.02	0.619	0.712
Genus Staphylococcus	0.86	0.02	0.712	0.712
Genus Streptococcus	1.49	0.04	0.055	0.247
Alphaproteobacteria unclassified genus	1.61	0.05	0.106	0.247

Table S5. Linear mixed model (LMM) results between treatments and within intervention and control groups for skin bacterial A) richness, B) relative abundance at order, and C) genus level. Data is presented as mean ± standard deviation (sd). LMM statistics are reported as t value, probability P value, and permuted p value (5,000 permutations).

**A) Richness**	**Intervention**				**Control**				**Between Treatments**			**Within Intervention Treatment**			**Within Control Treatment**		
**LMM: Day 0 vs Day 14**	**Day 0**		**Day 14**		**Day 0**		**Day 14**		**LMM: Day 0 vs Day 14**			**LMM: Day 0 vs Day 14**			**LMM: Day 0 vs Day 14**		
	**Mean**	**sd**	**Mean**	**sd**	**Mean**	**sd**	**Mean**	**sd**	**t value**	**p value**	**Perm.p**	**t value**	**p value**	**Perm.p**	**t value**	**p value**	**Perm.p**
Total bacterial	1196	370	1814	711	1262	376	1426	303	2.316	**0.021**	**0.026**	3.87	**0.0001**	**0.0002**	2.17	**0.03**	0.154
Phyla Actinobacteria	290	86	410	125	312	92	343	75	2.118	**0.034**	**0.038**	3.86	**0.0001**	**0.0002**	1.77	0.08	
Phyla Bacteroidetes	88	29	129	37	97	32	109	27	1.841	0.066		3.96	**0.0001**	**0.0002**	1.94	0.05	
Phyla Proteobacteria	257	80	372	113	265	76	304	60	2.037	**0.042**	**0.0498**	3.96	**0.0001**	**0.0002**	2.35	**0.02**	0.166
Phyla Firmicutes	424	132	611	183	451	120	505	103	2.168	**0.030**	**0.0354**	4.10	**0.0000**	**0.0004**	2.13	**0.03**	0.238
Class Bacilli	267	81	375	109	285	73	314	63	2.112	**0.035**	**0.0398**	3.88	**0.0001**	**0.0004**	1.76	0.08	
Class Betaproteobacteria	34	12	49	15	35	12	41	9	1.619	0.105		3.69	**0.0002**	**0.0005**	2.69	**0.01**	0.181
Class Gammaproteobacteria	180	56	261	79	184	53	208	44	2.119	**0.034**	**0.0408**	3.98	**0.0001**	**0.0002**	2.07	**0.04**	0.212
Class Alphaproteobacteria	36	13	52	17	38	11	45	9	1.708	0.088		3.68	**0.0002**	**0.0005**	2.29	**0.02**	0.084
Class Bacteroidia	60	18	87	27	66	21	75	20	1.770	0.077		3.88	**0.0001**	**0.0003**	1.90	0.06	
Class Clostridia	134	45	205	65	143	42	163	37	2.385	**0.017**	**0.0232**	4.56	**0.0000**	**0.0004**	2.42	**0.02**	0.220
Class Negativicutes	12	5	18	6	14	5	17	5	1.163	0.245		3.06	**0.0022**	**0.0033**	1.86	0.06	
Class Flavobacteriia	12	6	17	5	13	5	15	5	1.431	0.152		3.37	**0.0007**	**0.0013**	1.08	0.28	
**LMM: Day 0 vs Day 35**	**Day 0**		**Day 35**		**Day 0**		**Day 35**		**LMM: Day 0 vs Day 35**			**LMM: Day 0 vs Day 35**			**LMM: Day 0 vs Day 35**		
	**Mean**	**sd**	**Mean**	**sd**	**Mean**	**sd**	**Mean**	**sd**	**t value**	**p value**		**t value**	**p value**	**Perm.p**	**t value**	**p value**	**Perm.p**
Total bacterial	1196	370	1309	570	1262	376	1365	494	0.058	0.954		0.71	0.48		1.34	0.18	
Phyla Actinobacteria	290	86	309	130	312	92	329	118	0.051	0.960		0.61	0.54		0.94	0.35	
Phyla Bacteroidetes	88	29	103	47	97	32	106	41	0.360	0.719		1.41	0.16		1.28	0.20	
Phyla Proteobacteria	257	80	279	122	265	76	287	101	0.010	0.992		0.74	0.46		1.33	0.18	
Phyla Firmicutes	424	132	464	192	451	120	481	155	0.168	0.867		0.87	0.39		1.13	0.26	
Class Bacilli	267	81	291	123	285	73	301	98	0.201	0.841		0.86	0.39		1.00	0.32	
Class Betaproteobacteria	34	12	37	17	35	12	38	12	-0.026	0.979		0.74	0.46		1.27	0.20	
Class Gammaproteobacteria	180	56	195	85	184	53	198	72	0.042	0.966		0.74	0.46		1.24	0.22	
Class Alphaproteobacteria	36	13	38	18	38	11	43	17	-0.412	0.681		0.49	0.63		1.66	0.10	
Class Bacteroidia	60	18	71	31	66	21	72	26	0.470	0.639		1.50	0.13		1.15	0.25	
Class Clostridia	134	45	148	60	143	42	153	48	0.120	0.904		0.86	0.39		1.21	0.23	
Class Negativicutes	12	5	14	7	14	5	15	6	0.263	0.793		0.83	0.41		0.62	0.54	
Class Flavobacteriia	12	6	14	6	13	5	15	7	0.180	0.857		1.27	0.20		0.96	0.34	

B) Relative abundance at order level	Intervention				Control				Between Treatments			Within Intervention Treatment			Within Control Treatment		
	Day 0		Day 14		Day 0		Day 14		LMM: Day 0 vs Day 14			LMM: Day 0 vs Day 14			LMM: Day 0 vs Day 14		
LMM: Day 0 vs Day 14	Mean	sd	Mean	sd	Mean	sd	Mean	sd	t value	p value	Perm.p	t value	p value	Perm.p	t value	p value	Perm.p
Lactobacillales	400	38	444	51	406	30	419	29	2.01	0.044	0.047	3.81	0.000	0.001	1.72	0.085	0.079
Micropepsales	4	1	5	2	5	2	4	1	2.73	0.006	0.009	2.27	0.023	0.030	-1.84	0.066	0.090
Planctomycetales	22	5	25	6	21	5	19	4	2.56	0.010	0.012	2.49	0.013	0.019	-1.30	0.192	0.274
Veillonellales	51	8	61	9	53	6	55	6	2.26	0.024	0.025	3.81	0.000	0.000	0.80	0.421	0.421
Clostridiales	840	57	950	119	854	58	887	53	2.34	0.020	0.035	4.48	0.000	0.000	2.33	0.020	0.007
Micrococcales	604	44	672	84	620	44	642	40	2.04	0.041	0.038	3.87	0.000	0.001	2.40	0.016	0.027
Rhodobacterales	22	4	25	8	21	4	21	4	1.99	0.046	0.040	1.95	0.052	0.065	-0.24	0.814	0.805
Mycobacteriales	442	24	487	51	452	45	462	30	2.09	0.036	0.045	3.71	0.000	0.000	1.82	0.069	0.063
Bacteria unclassified	214	14	241	30	214	15	224	15	2.07	0.038	0.058	3.96	0.000	0.001	2.45	0.014	0.013
Pseudomonadales	170	13	186	18	175	20	177	17	2.03	0.042	0.059	3.87	0.000	0.001	0.73	0.464	0.375
Chitinophagales	36	5	44	9	37	6	40	6	1.88	0.060	0.067	4.10	0.000	0.000	1.96	0.050	0.076
Bacillales	1178	63	1283	118	1191	59	1227	53	1.89	0.058	0.075	3.54	0.000	0.002	2.00	0.046	0.048
Propionibacteriales	324	16	356	41	326	27	339	26	1.73	0.084	0.085	3.61	0.000	0.001	1.87	0.062	0.049
Burkholderiales	125	11	144	23	124	15	132	11	1.76	0.079	0.102	3.58	0.000	0.000	2.58	0.010	0.008
LMM: Day 0 vs Day 35	Day 0		Day 35		Day 0		Day 35		LMM: Day 0 vs Day 35			LMM: Day 0 vs Day 35			LMM: Day 0 vs Day 35		
	Mean	sd	Mean	sd	Mean	sd	Mean	sd	t value	p value	Perm.p	t value	p value	Perm.p	t value	p value	Perm.p
Lactobacillales	400	38	416	48	406	30	418	35	0.33	0.743	0.713	0.04	0.965	0.173	1.46	0.143	0.159
Micropepsales	4	1	4	1	5	2	4	2	1.50	0.134	0.138	0.27	0.788	0.941	-2.12	0.034	0.038
Planctomycetales	22	5	21	5	21	5	20	4	-0.05	0.959	0.894	-1.26	0.207	0.623	-0.36	0.716	0.792
Veillonellales	51	8	51	8	53	6	54	8	-0.22	0.829	0.800	0.89	0.374	0.898	0.17	0.864	0.781
Clostridiales	840	57	869	78	854	58	874	66	0.23	0.820	0.751	0.09	0.931	0.253	1.34	0.182	0.195
Micrococcales	604	44	616	61	620	44	622	55	0.42	0.677	0.762	1.66	0.098	0.501	0.46	0.648	0.492
Rhodobacterales	22	4	22	4	21	4	22	4	-0.51	0.609	0.651	0.60	0.549	0.857	1.56	0.118	0.117
Mycobacteriales	442	24	447	45	452	45	463	40	-0.35	0.723	0.754	0.07	0.941	0.702	1.33	0.185	0.149
Bacteria unclassified	214	14	214	21	214	15	218	13	-0.42	0.673	0.714	0.52	0.601	0.961	0.81	0.419	0.468
Pseudomonadales	170	13	175	17	175	20	180	16	0.06	0.950	0.854	-0.70	0.485	0.241	1.10	0.270	0.347
Chitinophagales	36	5	39	8	37	6	36	5	1.46	0.143	0.191	0.62	0.535	0.162	-0.76	0.446	0.609
Bacillales	1178	63	1207	132	1191	59	1210	77	0.28	0.780	0.713	-0.66	0.508	0.337	1.05	0.291	0.339
Propionibacteriales	324	16	328	30	326	27	333	32	-0.21	0.836	0.866	-0.53	0.597	0.653	1.19	0.234	0.277
Burkholderiales	125	11	129	15	124	15	122	11	1.09	0.278	0.282	1.45	0.148	0.433	-0.73	0.463	0.518

B) Relative abundance at genus level	Intervention				Control				Between Treatments			Within Intervention Treatment			Within Control Treatment		
	Day 0		Day 14		Day 0		Day 14		LMM: Day 0 vs Day 14			LMM: Day 0 vs Day 14			LMM: Day 0 vs Day 14		
**LMM: Day 0 vs Day 14**	**Mean**	**sd**	**Mean**	**sd**	**Mean**	**sd**	**Mean**	**sd**	**t value**	**p value**	**Perm.p**	**t value**	**p value**	**Perm.p**	**t value**	**p value**	**Perm.p**
Corynebacterium	357	22	390	38	367	37	372	26	2.121	**0.034**	**0.050**	3.632	**0.000**	**0.001**	1.061	0.289	0.275
Streptococcus	213	21	232	31	217	17	221	16	1.599	0.110	0.118	2.849	**0.004**	**0.005**	0.924	0.355	0.299
Chryseobacterium	18	3	23	6	18	4	18	3	2.746	**0.006**	**0.004**	3.683	**0.000**	0.003	-0.059	0.953	0.815
Gemmatimonas	2	1	5	3	3	1	3	1	2.950	**0.003**	**0.004**	3.565	**0.000**	0.001	0.365	0.715	0.832
Lawsonibacter	2	0	3	2	2	1	2	1	3.057	**0.002**	**0.004**	3.783	**0.000**	0.000	-0.201	0.841	0.982
Ruminococcus2	1	1	2	2	2	1	1	0	3.092	**0.002**	**0.005**	3.096	**0.002**	0.003	-1.401	0.161	0.245
Herminiimonas	2	0	4	2	2	0	2	1	2.745	**0.006**	**0.005**	3.497	**0.000**	0.001	1.558	0.119	0.128
Rhodobacteraceae unclassified	8	2	11	5	9	2	8	2	2.650	**0.008**	**0.006**	2.192	**0.028**	0.037	-1.630	0.103	0.081
Chitinophaga	1	1	2	1	1	1	1	0	2.764	**0.006**	**0.008**	2.098	**0.036**	0.040	-2.805	**0.005**	**0.011**
Rhodococcus	9	2	11	3	10	3	9	3	2.833	**0.005**	**0.008**	2.914	**0.004**	0.007	-1.083	0.279	0.416
Geobacillus	0	0	1	1	1	1	0	0	2.756	**0.006**	**0.009**	1.868	0.062	0.062	-2.105	**0.035**	**0.047**
Rubellimicrobium	2	1	4	3	2	1	3	1	2.666	**0.008**	**0.010**	3.880	**0.000**	0.000	0.749	0.454	0.436
Cuneatibacter	0	0	1	1	0	0	0	0	2.265	**0.024**	**0.011**	2.373	**0.018**	0.025	0.519	0.604	0.818
Veillonella	31	6	39	8	33	4	33	4	2.615	**0.009**	**0.012**	3.625	**0.000**	0.001	0.241	0.810	0.683
Tepidiphilus	1	1	0	0	0	0	0	1	-2.509	**0.012**	**0.012**	-2.224	**0.026**	0.034	1.151	0.250	0.242
Fusobacterium	17	4	18	4	14	3	18	3	-2.384	**0.017**	**0.013**	0.947	0.344	0.352	4.731	**0.000**	**0.000**
Lacunisphaera	0	0	0	1	0	1	0	0	2.470	**0.014**	**0.014**	1.263	0.207	0.234	-2.268	**0.023**	**0.022**
Clostridiales unclassified	66	11	78	16	69	9	68	9	2.595	**0.009**	**0.014**	3.572	**0.000**	0.001	0.190	0.849	0.584
Terrimonas	0	1	1	1	1	1	0	1	1.970	**0.049**	**0.016**	1.988	**0.047**	0.069	-0.792	0.429	0.112
Curvibacter	3	1	3	2	2	1	2	0	2.451	**0.014**	**0.020**	2.254	**0.024**	0.030	-1.545	0.122	0.256
Thermoactinomyces	1	0	2	1	1	0	1	0	2.442	**0.015**	**0.021**	2.305	**0.021**	0.032	-0.767	0.443	0.577
Methylocaldum	1	1	2	1	2	1	1	1	2.418	**0.016**	**0.021**	2.899	**0.004**	0.007	-0.610	0.542	0.622
Peptoanaerobacter	0	0	1	1	0	1	0	0	2.293	**0.022**	**0.021**	2.063	**0.039**	0.043	-1.074	0.283	0.295
Bacillales unclassified	233	17	264	29	242	18	247	17	2.492	**0.013**	**0.022**	4.122	**0.000**	0.000	1.150	0.250	0.183
Geobacter	1	0	1	1	1	0	1	0	2.277	**0.023**	**0.025**	2.015	**0.044**	0.059	-2.184	**0.029**	**0.015**
Roseomonas	1	1	1	1	1	1	1	1	-2.263	**0.024**	**0.025**	-1.906	0.057	0.076	1.251	0.211	0.180
Lactobacillaceae unclassified	24	6	30	8	25	6	26	6	2.080	**0.037**	**0.026**	3.425	**0.001**	0.002	-0.066	0.948	0.769
Planctomycetales unclassified	7	2	10	3	6	2	7	3	2.216	**0.027**	**0.029**	3.939	**0.000**	0.001	1.110	0.267	0.305
Rhizobiales unclassified	19	4	24	6	20	3	21	4	2.139	**0.032**	**0.029**	2.884	**0.004**	0.008	0.655	0.512	0.537
Cellvibrionales unclassified	1	0	1	1	1	0	1	0	2.076	**0.038**	**0.030**	2.018	**0.044**	0.048	-0.662	0.508	0.147

B) Relative abundance at genus level	Intervention				Control				Between Treatments			Within Intervention Treatment			Within Control Treatment		
	Day 0		Day 14		Day 0		Day 14		LMM: Day 0 vs Day 14			LMM: Day 0 vs Day 14			LMM: Day 0 vs Day 14		
**LMM: Day 0 vs Day 14**	**Mean**	**sd**	**Mean**	**sd**	**Mean**	**sd**	**Mean**	**sd**	**t value**	**p value**	**Perm.p**	**t value**	**p value**	**Perm.p**	**t value**	**p value**	**Perm.p**
Rothia	75	8	82	13	80	11	77	9	2.265	**0.024**	**0.030**	2.574	**0.010**	0.015	-0.583	0.560	0.776
Pseudoflavonifractor	1	0	2	1	1	0	1	0	1.986	**0.047**	**0.035**	1.776	0.076	0.089	-1.641	0.101	0.054
Micropepsaceae unclassified	3	1	4	2	4	2	3	1	2.213	**0.027**	**0.036**	1.696	0.090	0.102	-1.641	0.101	0.107
Candidimonas	1	1	2	2	1	0	1	1	2.159	**0.031**	**0.036**	2.575	**0.010**	0.015	0.163	0.870	0.871
Algoriella	1	0	2	1	1	0	1	0	2.084	**0.037**	**0.037**	2.181	**0.029**	0.039	0.348	0.728	0.778
Brevilactibacter	3	1	2	0	3	1	3	1	-2.227	**0.026**	**0.038**	-1.801	0.072	0.078	1.708	0.088	0.116
Thermoclostridium	0	0	0	1	0	1	0	0	2.130	**0.033**	**0.038**	2.234	**0.026**	0.027	-1.399	0.162	0.195
Brochothrix	7	2	9	4	8	3	8	3	2.155	**0.031**	**0.039**	2.263	**0.024**	0.029	-0.496	0.620	0.712
Propionibacteriaceae unclassifie	10	2	13	4	10	2	11	4	2.069	**0.039**	**0.039**	3.903	**0.000**	0.001	0.920	0.358	0.398
Ezakiella	1	1	2	2	1	1	1	1	2.041	**0.041**	**0.041**	1.942	0.052	0.062	-0.787	0.431	0.405
Faecalitalea	1	0	1	0	1	0	1	1	-1.965	**0.049**	**0.042**	-2.072	**0.038**	0.048	1.237	0.216	0.206
Micrococcaceae unclassified	230	19	263	33	237	15	252	23	2.044	**0.041**	**0.042**	4.834	**0.000**	0.000	3.596	**0.000**	**0.001**
Neisseria	20	6	24	6	20	4	20	5	1.924	0.054	**0.047**	3.022	**0.003**	0.004	-0.194	0.846	0.780
Eisenbergiella	1	1	1	1	3	2	2	2	1.610	0.107	**0.048**	0.518	0.604	0.637	-2.532	**0.011**	**0.009**
Mesobacillus	0	0	0	1	0	1	0	0	2.108	**0.035**	0.050	1.785	0.074	0.084	-1.468	0.142	0.155
Arcanobacterium	0	0	0	1	0	1	0	0	1.894	0.058	0.052	1.194	0.232	0.256	-1.591	0.112	0.098
Ectothiorhodospiraceae unclassi	0	1	0	0	0	0	0	1	-1.900	0.057	0.052	-1.547	0.122	0.127	1.380	0.167	0.169
Luteimonas	4	2	5	3	4	2	4	2	1.964	**0.050**	0.052	1.482	0.138	0.161	-2.164	**0.030**	0.054
Actinomycetospora	2	2	1	1	1	1	2	2	-2.263	**0.024**	0.053	-1.247	0.212	0.224	2.553	**0.011**	**0.036**
Skermanella	1	0	2	1	1	1	1	1	1.946	0.052	0.056	2.529	**0.011**	0.014	-0.371	0.711	0.701
Bacteria unclassified	214	14	241	30	214	15	224	15	2.074	**0.038**	0.058	3.957	**0.000**	0.001	2.448	**0.014**	**0.017**
Thermomonosporaceae unclassi	3	2	2	3	3	2	4	2	-1.618	0.106	0.066	-0.985	0.325	0.336	1.383	0.167	0.111
Duganella	6	2	8	4	5	1	5	2	1.804	0.071	0.066	2.427	**0.015**	0.023	0.262	0.793	0.905
Acidimicrobiales unclassified	5	2	4	1	5	2	5	1	-1.670	0.095	0.067	-3.077	**0.002**	0.003	0.217	0.828	0.562
Ruminococcaceae unclassified	132	13	156	21	133	12	144	13	1.967	**0.049**	0.067	4.840	**0.000**	0.000	3.741	**0.000**	**0.000**
Schaalia	31	5	35	6	29	4	30	4	1.705	0.088	0.068	2.201	**0.028**	0.041	0.076	0.939	0.946
Parvibaculum	1	0	1	0	1	0	1	0	-1.910	0.056	0.068	-3.955	**0.000**	0.001	-2.704	**0.007**	**0.007**
Sphingopyxis	1	0	1	0	1	0	1	0	-1.910	0.056	0.069	-3.955	**0.000**	0.000	-2.704	**0.007**	**0.007**
Massilimicrobiota	2	0	2	0	2	0	2	0	-1.910	0.056	0.069	-3.955	**0.000**	0.001	-2.704	**0.007**	**0.007**
Staphylococcus	265	21	299	38	269	20	283	25	1.861	0.063	0.072	3.840	**0.000**	0.000	2.238	**0.025**	**0.028**

B) Relative abundance at genus level	Intervention				Control				Between Treatments			Within Intervention Treatment			Within Control Treatment		
	Day 0		Day 35		Day 0		Day 35		LMM: Day 0 vs Day 35			LMM: Day 0 vs Day 35			LMM: Day 0 vs Day 35		
LMM: Day 0 vs Day 35	Mean	sd	Mean	sd	Mean	sd	Mean	sd	t value	p value	Perm.p	t value	p value	Perm.p	t value	p value	Perm.p
Corynebacterium	357	22	362	34	367	37	374	33	-0.088	0.930	0.992	0.555	0.579	0.578	0.765	0.444	0.411
Streptococcus	213	21	217	23	217	17	224	19	-0.298	0.766	0.840	0.656	0.512	0.517	1.327	0.185	0.222
Chryseobacterium	18	3	19	3	18	4	19	4	0.233	0.816	0.865	0.876	0.381	0.419	0.815	0.415	0.366
Gemmatimonas	2	1	3	1	3	1	3	1	0.041	0.967	0.923	0.410	0.682	0.680	0.623	0.533	0.402
Lawsonibacter	2	0	2	1	2	1	2	1	0.456	0.649	0.628	1.718	0.086	0.097	2.164	0.030	0.060
Ruminococcus2	1	1	2	1	2	1	2	1	-0.669	0.503	0.543	0.333	0.739	0.734	1.378	0.168	0.240
Herminiimonas	2	0	2	1	2	0	2	1	1.014	0.311	0.309	0.986	0.324	0.344	-0.329	0.742	0.820
Rhodobacteraceae unclassified	8	2	9	2	9	2	8	2	0.373	0.709	0.639	0.159	0.874	0.884	-0.533	0.594	0.535
Chitinophaga	1	1	1	1	1	1	1	0	1.046	0.295	0.333	0.062	0.950	0.952	-2.182	0.029	0.037
Rhodococcus	9	2	9	3	10	3	8	2	1.323	0.186	0.205	0.172	0.864	0.872	-1.866	0.062	0.115
Geobacillus	0	0	1	1	1	1	0	0	2.480	**0.013**	0.016	1.249	0.212	0.220	-2.440	0.015	0.017
Rubellimicrobium	2	1	2	1	2	1	3	1	-0.324	0.746	0.766	0.165	0.869	0.874	0.819	0.413	0.466
Cuneatibacter	0	0	0	0	0	0	0	0	0.038	0.970	0.880	-0.081	0.936	0.944	-0.368	0.713	0.478
Veillonella	31	6	32	6	33	4	33	6	0.263	0.793	0.866	0.600	0.549	0.555	0.333	0.739	0.572
Tepidiphilus	1	1	0	0	0	0	0	1	-1.882	0.060	0.069	-1.228	0.219	0.234	1.618	0.106	0.131
Fusobacterium	17	4	16	4	14	3	16	3	-2.175	**0.030**	0.029	-1.205	0.228	0.254	1.997	0.046	0.050
Lacunisphaera	0	0	0	1	0	1	0	0	2.387	**0.017**	0.018	1.227	0.220	0.237	-2.209	0.027	0.027
Clostridiales unclassified	66	11	72	13	69	9	73	11	0.412	0.680	0.639	1.828	0.068	0.076	1.657	0.098	0.107
Terrimonas	0	1	0	1	1	1	0	1	0.688	0.491	0.732	0.081	0.936	0.942	-0.952	0.341	0.591
Curvibacter	3	1	2	1	2	1	2	1	0.082	0.934	0.994	-0.269	0.788	0.798	-0.691	0.490	0.624
Thermoactinomyces	1	0	1	1	1	0	1	0	1.687	0.092	0.098	1.527	0.127	0.146	-0.774	0.439	0.485
Methylocaldum	1	1	1	1	2	1	1	1	1.487	0.137	0.116	1.656	0.098	0.107	-0.518	0.605	0.532
Peptoanaerobacter	0	0	0	0	0	1	0	1	0.305	0.760	0.746	0.000	1.000	1.000	-0.403	0.687	0.655
Bacillales unclassified	233	17	237	35	242	18	248	19	-0.275	0.784	0.874	0.408	0.683	0.675	1.117	0.264	0.347
Geobacter	1	0	1	0	1	0	1	0	0.191	0.848	0.902	0.049	0.961	0.961	-1.152	0.249	0.330
Roseomonas	1	1	0	0	1	1	1	1	-1.878	0.060	0.072	-2.978	0.003	0.006	-0.527	0.598	0.598
Lactobacillaceae unclassified	24	6	27	8	25	6	27	7	0.213	0.831	0.859	1.679	0.093	0.099	1.519	0.129	0.172
Planctomycetales unclassified	7	2	7	2	6	2	7	3	-0.543	0.587	0.450	0.125	0.901	0.894	0.999	0.318	0.201
Rhizobiales unclassified	19	4	22	6	20	3	20	4	1.017	0.309	0.334	1.495	0.135	0.154	0.456	0.648	0.496
Cellvibrionales unclassified	1	0	1	0	1	0	1	0	-0.422	0.673	0.753	-0.106	0.916	0.921	0.896	0.370	0.706

B) Relative abundance at genus level	Intervention				Control				Between Treatments			Within Intervention Treatment			Within Control Treatment		
	Day 0		Day 35		Day 0		Day 35		LMM: Day 0 vs Day 35			LMM: Day 0 vs Day 35			LMM: Day 0 vs Day 35		
LMM: Day 0 vs Day 35	Mean	sd	Mean	sd	Mean	sd	Mean	sd	t value	p value	Perm.p	t value	p value	Perm.p	t value	p value	Perm.p
Rothia	75	8	77	10	80	11	80	9	0.607	0.544	0.533	0.853	0.393	0.403	0.052	0.959	0.908
Pseudoflavonifractor	1	0	1	0	1	0	1	0	-0.038	0.969	0.946	-0.204	0.838	0.851	-1.200	0.230	0.348
Micropepsaceae unclassified	3	1	3	1	4	2	3	1	1.289	0.197	0.185	-0.128	0.898	0.909	-2.347	0.019	0.022
Candidimonas	1	1	1	1	1	0	1	1	0.274	0.784	0.781	0.800	0.424	0.424	0.853	0.394	0.426
Algoriella	1	0	1	0	1	0	1	0	0.138	0.891	0.908	-0.001	0.999	0.999	-0.774	0.439	0.469
Brevilactibacter	3	1	2	1	3	1	3	1	-0.927	0.354	0.293	-1.260	0.208	0.217	0.169	0.865	0.747
Thermoclostridium	0	0	0	0	0	1	0	1	0.166	0.868	0.886	0.000	1.000	1.000	-0.345	0.730	0.705
Brochothrix	7	2	8	3	8	3	7	2	1.376	0.169	0.159	0.602	0.547	0.553	-1.677	0.093	0.072
Propionibacteriaceae unclassifie	10	2	10	3	10	2	12	3	-0.794	0.427	0.436	0.677	0.499	0.496	1.740	0.082	0.064
Ezakiella	1	1	2	1	1	1	2	1	0.102	0.919	0.911	0.786	0.432	0.440	0.958	0.338	0.407
Faecalitalea	1	0	1	0	1	0	1	0	-0.068	0.946	0.981	-0.389	0.697	0.711	-0.085	0.932	0.968
Micrococcaceae unclassified	230	19	234	20	237	15	237	20	0.495	0.621	0.724	0.636	0.525	0.534	0.270	0.787	0.616
Neisseria	20	6	20	4	20	4	21	7	-0.396	0.692	0.622	-0.148	0.882	0.884	0.491	0.624	0.521
Eisenbergiella	1	1	2	2	3	2	2	2	2.016	**0.044**	0.089	0.723	0.470	0.501	-2.294	0.022	0.030
Mesobacillus	0	0	0	0	0	1	0	1	0.326	0.745	0.737	0.000	1.000	1.000	-0.361	0.718	0.656
Arcanobacterium	0	0	0	0	0	1	0	0	0.720	0.472	0.495	-0.087	0.931	0.938	-1.422	0.155	0.142
Ectothiorhodospiraceae unclassi	0	1	0	0	0	0	0	1	-1.851	0.064	0.063	-1.547	0.122	0.132	1.251	0.211	0.225
Luteimonas	4	2	4	2	4	2	4	2	0.650	0.516	0.485	-0.163	0.870	0.880	-1.886	0.059	0.047
Actinomycetospora	2	2	1	1	1	1	1	1	-0.137	0.891	0.740	-0.553	0.580	0.588	-0.152	0.879	0.871
Skermanella	1	0	1	0	1	1	1	1	-0.253	0.800	0.802	0.096	0.923	0.922	0.585	0.558	0.575
Bacteria unclassified	214	14	214	21	214	15	218	13	-0.422	0.673	0.714	0.053	0.958	0.961	0.809	0.419	0.465
Thermomonosporaceae unclassi	3	2	3	3	3	2	3	2	-0.478	0.633	0.649	0.096	0.924	0.926	0.816	0.414	0.491
Duganella	6	2	5	2	5	1	5	2	-0.707	0.480	0.560	-0.570	0.569	0.564	0.607	0.544	0.751
Acidimicrobiales unclassified	5	2	5	1	5	2	5	2	-1.252	0.211	0.244	-1.340	0.180	0.193	0.989	0.322	0.349
Ruminococcaceae unclassified	132	13	141	14	133	12	136	10	1.013	0.311	0.294	1.924	0.054	0.065	1.026	0.305	0.267
Schaalia	31	5	31	6	29	4	29	3	0.312	0.755	0.647	0.194	0.846	0.850	-0.336	0.737	0.608
Parvibaculum	1	0	1	0	1	0	1	0	0.078	0.938	0.940	-0.846	0.398	0.410	-1.629	0.103	0.092
Sphingopyxis	1	0	1	0	1	0	1	0	0.078	0.938	0.940	-0.846	0.398	0.402	-1.629	0.103	0.103
Massilimicrobiota	2	0	2	0	2	0	2	0	0.078	0.938	0.941	-0.846	0.398	0.408	-1.629	0.103	0.098
Staphylococcus	265	21	279	33	269	20	278	23	0.550	0.582	0.513	1.613	0.107	0.114	1.299	0.194	0.222

Table S6. BLAST results and Linear mixed model (LMM) results between treatments at day 14 and 35. Data is presented as mean ± standard deviation (sd). LMM statistics are reported as t value and probability P value.

		Intervention						Control									
		Day 0		Day 14		Day 35		Day 0		Day 14		Day 35		LMM Day 14		LMM Day 35	
**ASV**	**BLAST result: Percent identity and description**	mean	sd	mean	sd	mean	sd	mean	sd	mean	sd	mean	sd	t value	p value	t value	p value
**ASV_010301**	99.60% Uncultured bacterium clone ncm75h02c1 16S ribosomal RNA gene, partial sequence	1.36	0.24	1.97	1.43	1.31	0.21	1.43	0.27	1.26	0.10	1.41	0.26	2.71	0.007	-0.09	0.926
**ASV_014026**	99.62%Uncultured bacterium clone OTU_2723 16S ribosomal RNA gene, partial sequence	1.09	0.05	1.32	0.84	1.07	0.09	1.14	0.25	1.04	0.05	1.13	0.25	1.98	0.048	-0.04	0.969
**ASV_065844**	99.21% Uncultured bacterium clone 45 16S ribosomal RNA gene, partial sequence	0.86	0.09	1.48	1.22	0.97	0.60	0.89	0.11	0.83	0.04	0.86	0.10	2.55	0.011	0.48	0.628
**ASV_017155**	99.60% Uncultured Mobiluncus sp. partial 16S rRNA gene, isolate 207T_20009	0.96	0.42	1.38	1.07	0.89	0.27	0.97	0.37	0.89	0.27	0.86	0.10	2.02	0.043	0.15	0.880
**ASV_018499**	99.21% Uncultured bacterium clone 751 16S ribosomal RNA gene, partial sequence	1.01	0.05	1.51	1.08	1.10	0.46	1.00	0.06	0.96	0.05	1.11	0.59	2.15	0.031	-0.10	0.919
**ASV_019998**	100% Uncultured bacterium clone OTU_876 16S ribosomal RNA gene, partial sequence	1.11	0.23	1.67	1.04	1.05	0.19	1.08	0.21	1.18	0.11	1.08	0.22	2.15	0.031	-0.24	0.810
**ASV_020631**	100% Uncultured bacterium partial 16S rRNA gene	0.90	0.05	1.40	1.27	0.98	0.39	0.90	0.06	0.85	0.05	0.87	0.07	2.12	0.034	0.39	0.694
**ASV_021055**	99.21% Uncultured bacterium clone MPG-bac41 16S ribosomal RNA gene, partial sequence	0.94	0.05	1.28	1.01	0.92	0.09	0.94	0.06	0.89	0.05	0.91	0.07	1.97	0.049	0.02	0.981
**ASV_022295**	99.21% Halomonas sp. strain SA-66 16S ribosomal RNA gene, partial sequence	2.48	0.12	2.82	0.80	2.51	0.16	2.52	0.06	2.48	0.05	2.50	0.07	2.39	0.017	0.36	0.721
**ASV_026398**	98.42% Acinetobacter septicus strain AS002 16S ribosomal RNA gene, partial sequence	0.74	0.05	1.12	0.98	0.80	0.25	0.73	0.06	0.69	0.05	0.71	0.07	2.23	0.026	0.46	0.644
**ASV_029682**	99.21% uncultured Staphylococcus sp.	0.78	0.05	1.46	1.27	1.03	0.81	0.90	0.53	0.73	0.05	0.78	0.16	2.79	0.005	1.18	0.238

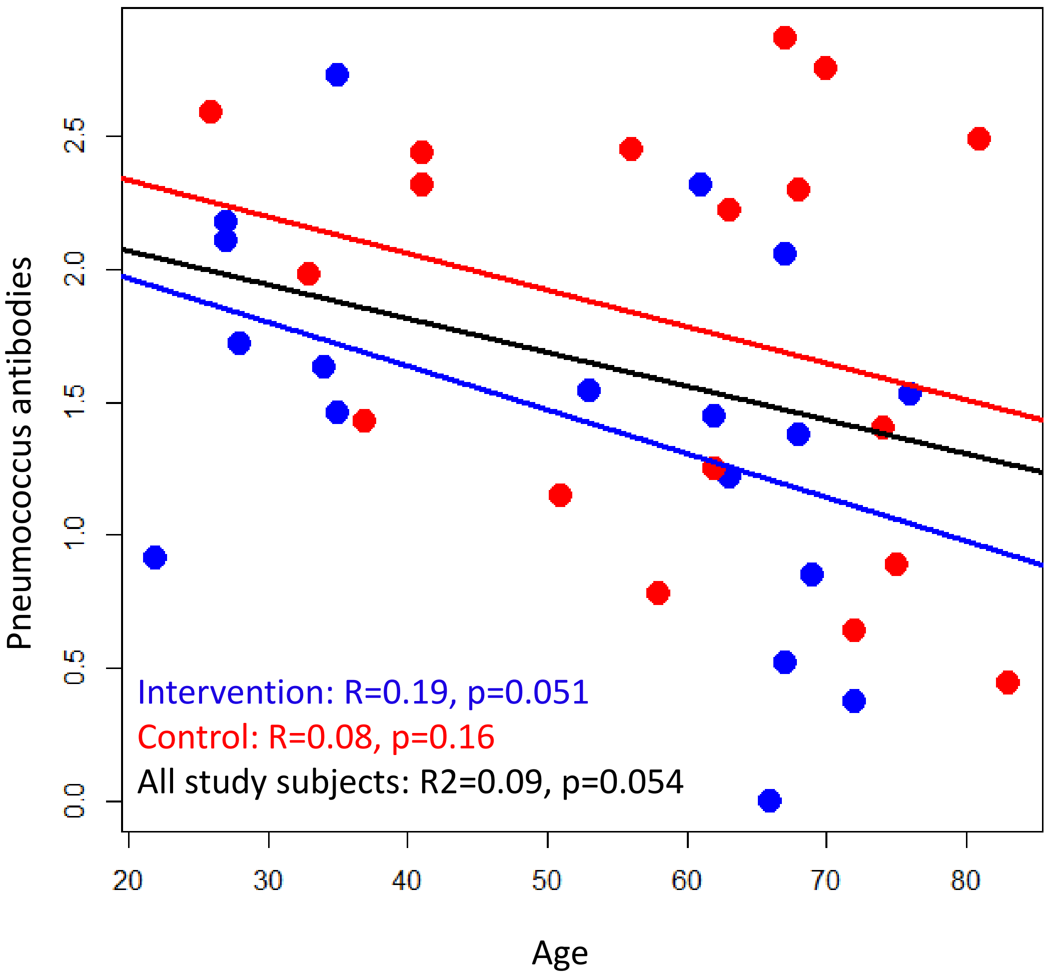

Figure S1. Correlation between age and pneumococcal antibody levels.

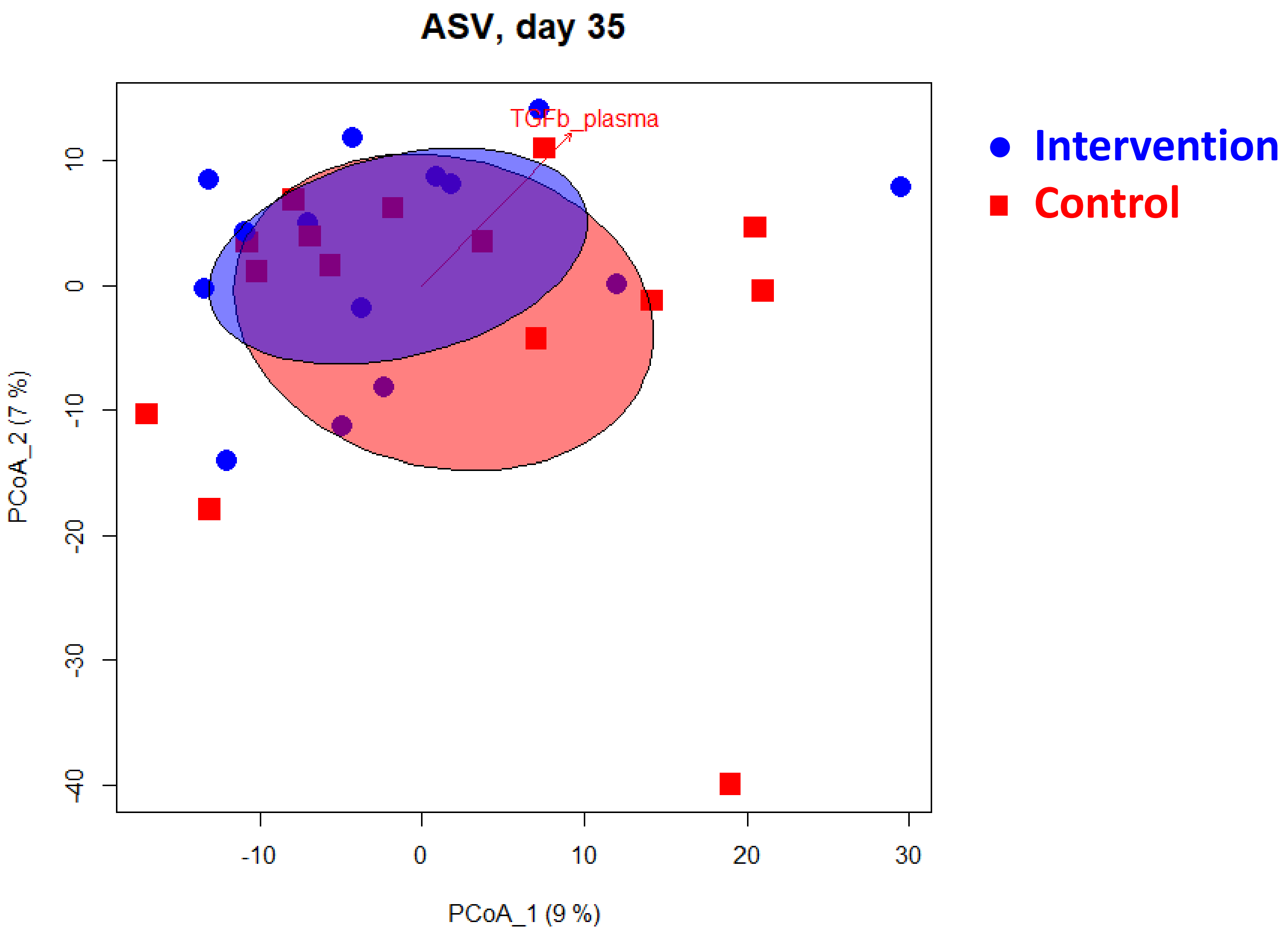

Figure S2. Beta diversity plot. Post hoc fitting of TGF-β on PCoA ordination (Envfit) for gut bacteria on day 35 at ASV level.
